# The IgG4 hinge with CD28 transmembrane domain improves V_H_H-based CAR T cells targeting a membrane-distal epitope of GPC1 in pancreatic cancer

**DOI:** 10.1038/s41467-023-37616-4

**Published:** 2023-04-08

**Authors:** Nan Li, Alex Quan, Dan Li, Jiajia Pan, Hua Ren, Gerard Hoeltzel, Natalia de Val, Dana Ashworth, Weiming Ni, Jing Zhou, Sean Mackay, Stephen M. Hewitt, Raul Cachau, Mitchell Ho

**Affiliations:** 1 Laboratory of Molecular Biology, Center for Cancer Research, National Cancer Institute, National Institutes of Health, Bethesda, MD 20892 USA; 2 Center for Molecular Microscopy, Center for Cancer Research, National Cancer Institute, National Institutes of Health, Frederick, MD 21702 USA; 3 IsoPlexis Corporation, Branford, CT 06405 USA; 4 Laboratory of Pathology, Center for Cancer Research, National Cancer Institute, National Institutes of Health, Bethesda, MD 20892 USA; 5 Integrated Data Science Section, Research Technologies Branch, National Institute of Allergy and Infectious Diseases, National Institutes of Health, Bethesda, MD 20892 USA

**Keywords:** Preclinical research, Translational research, Pancreatic cancer

## Abstract

Heterogeneous antigen expression is a key barrier influencing the activity of chimeric antigen receptor (CAR) T cells in solid tumors. Here, we develop CAR T cells targeting glypican-1 (GPC1), an oncofetal antigen expressed in pancreatic cancer. We report the generation of dromedary camel V_H_H nanobody (D4)-based CAR T cells targeting GPC1 and the optimization of the hinge (H) and transmembrane domain (TM) to improve activity. We find that a structurally rigid IgG4H and CD28TM domain brings the two D4 fragments in proximity, driving CAR dimerization and leading to enhanced T-cell signaling and tumor regression in pancreatic cancer models with low antigen density in female mice. Furthermore, single-cell-based proteomic and transcriptomic analysis of D4-IgG4H-CD28TM CAR T cells reveals specific genes (e.g., *HMGB1*) associated with high T-cell polyfunctionality. This study demonstrates the potential of V_H_H-based CAR T for pancreatic cancer therapy and provides an engineering strategy for developing potent CAR T cells targeting membrane-distal epitopes.

## Abstract (web summary)

Glypican-1 (GPC1) expression is elevated in pancreatic cancer and has been exploited as a therapeutic target. Here the authors report the development of V_H_H nanobody-based CAR-T cells targeting GPC1, showing anti-tumor activity in pancreatic cancer preclinical models.

## Introduction

Promising responses following CD19 chimeric antigen receptor (CAR) T-cell therapy in patients with relapsed and refractory B cell malignancies have led to the approval of four CD19 CAR T-cell products by the Food and Drug Administration (FDA)^1–3^. However, emerging follow-up data demonstrates that only 30–50% of patients experience long-term disease control^4,5^. To improve the response rate in B cell malignancies and translate the success of CAR T cells to solid tumors, the optimization of this class of therapeutics is required. Several studies have demonstrated that CAR hinge (H) and transmembrane (TM) domains influence CAR T-cell activity^6–8^. Additionally, effective antigen recognition depends on the epitope position and the length of the CAR extracellular spacer^9–11^. Thus, the design of CARs needs to consider both the epitope within the target and the nature and length of the spacer and TM regions.

Glypican-1 (GPC1) is a glycosylphosphatidylinositol-anchored cell surface protein. It is mainly expressed in the neural and skeletal systems during embryonic development and is expressed at low levels in adult tissues^12^. Multiple studies have shown that GPC1 expression is elevated in pancreatic cancer, both in cancer cells and the adjacent fibroblasts, whereas its expression is rarely found in normal pancreas^13,14^. Anti-GPC1 monoclonal antibodies have been utilized to develop an antibody-drug-conjugate (ADC), immunotoxin, bispecific T-cell engager (BiTE), and CAR T cells against GPC1-positive tumor cells and their antitumor efficacy has been demonstrated in preclinical models^15–20^. Moreover, miltuximab, a chimeric anti-GPC1 antibody, was shown to be safe and well tolerated when radiolabeled in a clinical trial^21^.

Our attention has been drawn to the various expression levels of GPC1 in pancreatic cancer. A recent study showed that 51.4% had weak, 35.1% had moderate, and 13.5% had strong staining of GPC1 in positively stained pancreatic tumor tissues^22^. Antigen density has emerged as a major factor influencing the activity of CAR T cells^23,24^. CAR T-cell potency is highly dependent on target antigen expression, and CARs often fail to exert their antitumor activities when antigen expression is low or below a certain threshold.

In this work, we isolate a dromedary camel V_H_H single-domain antibody (also known as nanobody) named D4 and a monoclonal antibody (mAb) named HM2 recognizing membrane-distal and membrane-proximal epitopes of GPC1, respectively. We demonstrate that replacing a lengthy CD8H with a short IgG4H significantly improves D4 CAR T-cell activity. Furthermore, by investigating the impact of hinge (CD8 and IgG4) and TM (CD8 and CD28) on D4 CAR T cells, we discover that IgG4H-CD28TM mediated D4 CAR dimerization, leading to enhanced T-cell signaling and tumor regression in pancreatic cancer models with low antigen density. Furthermore, the high polyfunctional CD8^+^ D4-IgG4H-CD28TM CAR T cells secrete more IL-2 and IL-7 and show increased expression of genes (e.g., *ID1*) that are important for T-cell long-term persistence. This work provides a strategy to optimize the recognition of a membrane-distal epitope and improves the function of nanobody-based CAR T cells in solid tumors.

## Results

### Isolation of high affinity GPC1-specific antibodies that recognize membrane-distal and membrane-proximal epitopes

**Fig. 1:**
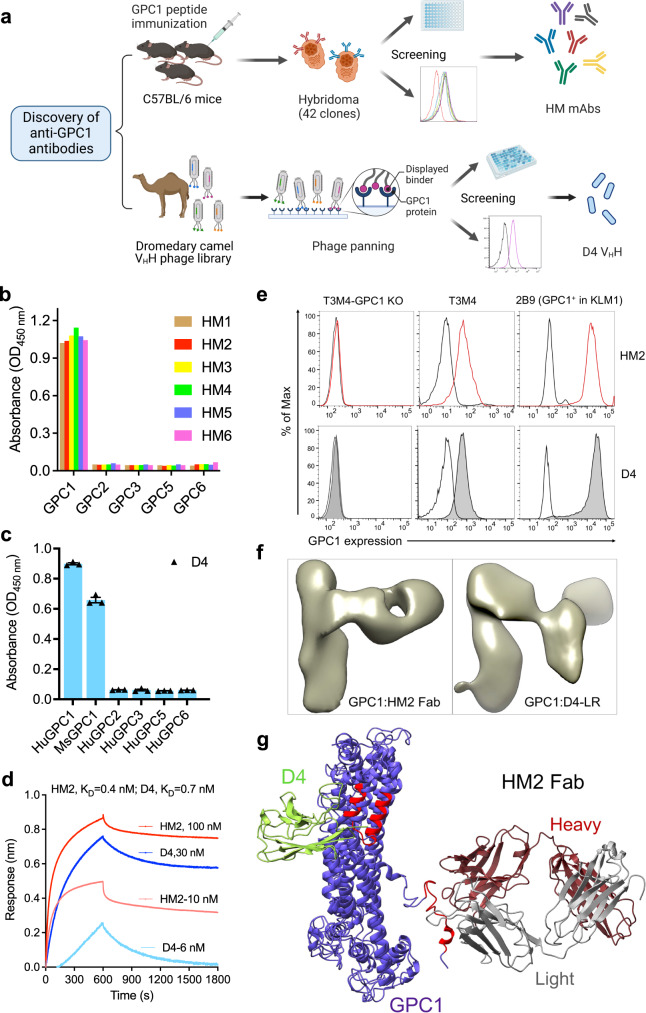
Isolation of GPC1-specific antibodies using hybridoma technology and phage display technology. **a** Discovery of anti-GPC1 monoclonal antibody (mAb) HM2 and camel V_H_H nanobody D4. The illustration was created with BioRender.com. **b** Six mouse mAbs (HM1 to HM6) at 1 μg/ml from three parental clones only bound to human GPC1, but not other human glypican members by ELISA. *n* = 1 independent experiment. **c** Monoclonal phage ELISA analysis of reactivity of D4 to human/mouse GPC1 as well as other human glypican members. *n* = 3 independent experiments. **d** Octet kinetic analysis for the interaction between HM2 or D4 and human GPC1. *n* = 1 independent experiment. **e** Cell surface GPC1 expression in GPC1-negative A431 cells, GPC1-positive pancreatic cancer cells T3M4, GPC1-knockout (KO) T3M4 cells, as well as GPC1-overexpressing cells 2B9. Data are representative of two independent experiments. White peaks represent the cell surface staining with isotype control. Red colored peaks and shaded gray peaks represent the cell surface staining of GPC1 using HM2 and D4, respectively, at 10 μg/ml. **f** Enlarged views of a 2D class average of GPC1 in complex with HM2 Fab and GPC1 in complex with D4-LR. **g** A ribbon diagram of the GPC1/HM2 Fab/D4 model. D4 and HM2 recognize the membrane-distal and membrane-proximal epitopes of GPC1, respectively, which are on the opposite sides of GPC1. The epitopes are highlighted in red color. Data are represented as mean ± SEM. Source data are provided as a Source Data file.

Although glypican members share ~25% amino acid similarity, their C-terminal regions close to the cell membrane have low sequence similarity based on our sequence analysis (Supplementary Fig. 1). To isolate mAbs targeting membrane-proximal GPC1-specific epitopes, we began by immunizing mice with the C-lobe region of GPC1 (Fig. 1a). Six mAbs (HM1 through HM6) were selected from 42 hybridoma clones and all specifically reacted to human GPC1 (Fig. 1b). Although they bound to GPC1 expressed on T3M4 pancreatic cancer cell line with similar affinity (Supplementary Fig. 2a), the HM2 clone was chosen for the following studies as it showed the highest protein production yield among all the mAbs (Supplementary Table 1). To identify a nanobody specific for GPC1, we constructed and screened a phage-displayed dromedary camel V_H_H nanobody library (Fig. 1a). After three rounds of panning, phage pools exhibited enhanced binding to GPC1 (Supplementary Fig. 2b). One V_H_H nanobody named D4 was identified by monoclonal phage ELISA and sequencing (Fig. 1c). D4 specifically recognized human GPC1 but not other human glypican members. Interestingly, it also cross-reacts with mouse GPC1. The kinetic analysis using Octet revealed that both HM2 and D4 bound to human GPC1 stably with high affinity (Fig. 1d). The K_D_ values of HM2 and D4 for GPC1 protein were 0.4 nM and 0.7 nM, respectively. We also examined the binding of HM2 and D4 to GPC1 on live cells by flow cytometry. Both antibodies bound equally well to GPC1-expressing T3M4 and KLM1 pancreatic cancer cells, GPC1-overexpressing A431 cells (H8), and GPC1-overexpressing KLM1 cells (2B9) (Fig. 1e and Supplementary Fig. 2c). Conversely, no binding was found with GPC1-negative A431 cells and GPC1-knockout (KO) T3M4 cells, suggesting that both bindings are antigen-specific. Taken together, we successfully identified a mouse mAb (HM2), and a camel nanobody (D4) specific for GPC1.

To identify the epitopes of HM2 and D4, we made a GPC1 peptide library that comprises 18 amino acid peptides with nine amino acid overlap. The sequences are listed in Supplementary Table 2. We found that HM2 specifically reacted with peptide 53, while D4 recognized epitopes comprising both peptides 14 and 15 (Supplementary Fig. 2d). Furthermore, we applied negative stain electron microscopy (EM) to analyze the structure of the GPC1:HM2 antibody-binding fragment (Fab) complex and GPC1:D4-LR complex (Fig. 1f). As the small size of the D4 V_H_H limits its application in EM, we used D4-LR which is an immunotoxin that contains domain III of *Pseudomonas* Exotoxin A (PE)^20^ here. The EM images and final three-dimensional (3D) reconstruction models revealed that D4 and HM2 Fab bound to the membrane-distal and membrane-proximal epitopes of GPC1, respectively (Fig. 1g). The binding epitopes of D4 and HM2 are on the opposite sides of GPC1.

### GPC1 expression is elevated in pancreatic cancer

To assess GPC1 levels in pancreatic cancer, we performed RT-PCR and western blot using a panel of pancreatic cancer cell lines and a normal human pancreatic duct epithelial cell line (hTERT-HPNE). GPC1 mRNA (Supplementary Fig. 3a) and protein (Supplementary Fig. 3b) levels were appreciably higher in nearly 90% of pancreatic cancer cell lines compared with normal pancreatic duct epithelial cells. Next, we evaluated the altered GPC1 expression in pancreatic cancer by performing immunohistochemistry (IHC) with the HM2 antibody. As shown in Supplementary Fig. 3c, we found elevated GPC1 expression in pancreatic tumor tissues from low-intermediate to high levels, but absence of GPC1 labeling in normal pancreas. Among 60 specimens of pancreatic cancer, 3 cases (5%) showed high GPC1 expression (IHC score of +3), 56 cases (93%) showed low to intermediate levels of staining (IHC scores of +1 or +2), and no immunoreactivity was observed in 1 case (2%) (Supplementary Fig. 4 and Supplementary Table 3). Moreover, we detected GPC1 expression in fibroblasts surrounding the cancer cells, which is consistent with previous reports^14^. In the same tissue microarray (Supplementary Fig. 4), 2 cases (3%) exhibited strong GPC1 staining (IHC score of +3) in fibroblast, 52 cases (87%) with low to intermediate levels of staining (IHC scores of +1 or +2), and no immunoreactivity was observed in 6 case (10%). Normal tissue adjacent to the tumor (referred to as NAT) is in an intermediate and pre-neoplastic state between healthy and tumor tissue^25^. Here, we found that GPC1 expression was increased in 4 of 6 NAT specimens (Supplementary Figs. 3d and 4), indicating GPC1 may play a role in pancreatic cancer tumorigenesis and/or progression.

### GPC1-targeted CAR T cells specifically kill GPC1-positive cancer cells in vitro and in vivo

**Fig. 2:**
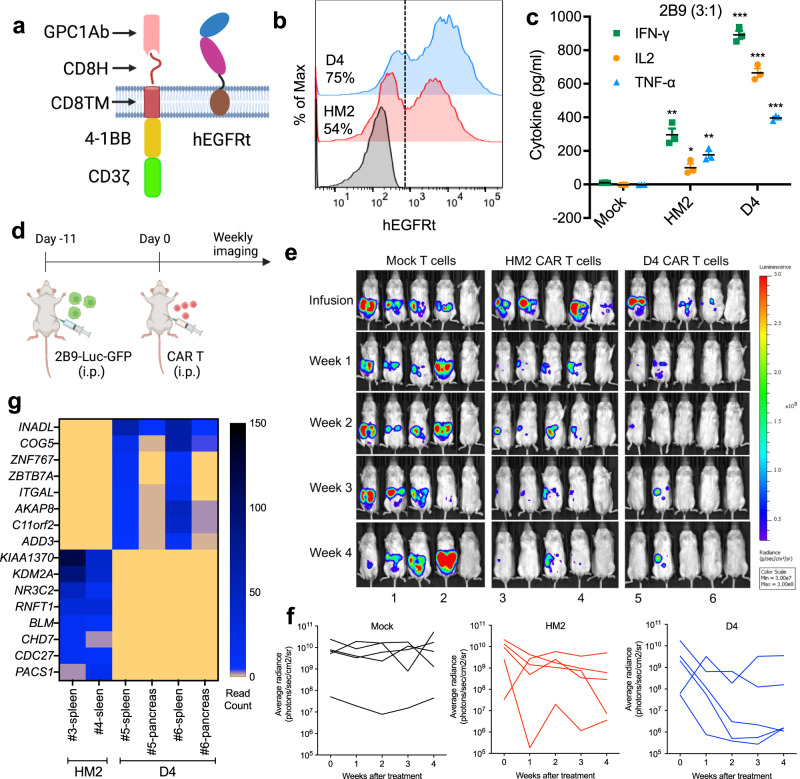
GPC1-targeted CAR T cells eradicate tumors in the 2B9 intraperitoneal dissemination xenograft mouse model. **a** Schematic of the GPC1-targeted CAR construct. The illustration was created with BioRender.com. **b** CARs expression on T cells were analyzed using flow cytometry by detection of hEGFRt expression. Data are representative of two independent experiments. **c** The secretion of IFN-γ, IL-2 and TNF-α in the CAR T and 2B9 co-cultured supernatant at E:T ratio of 3:1. *n* = 3 independent experiments. *p = 0.011, **p < 0.01, ***p < 0.001, two-tailed unpaired Student’s *t* test. **d** Experiment schematic (created with BioRender.com). 2B9 tumor-bearing NSG mice were treated with i.p. injection of 10 million mock T cells, HM2 CAR T cells and D4 CAR T cells on day 11 after tumor cell inoculation. *n* = 5 mice/group. **e** HM2 and D4 CAR T cells regressed established 2B9 xenografts in 4 of 5 mice in each group. **f** Tumor bioluminescence as photons per second in the Mock, HM2, and D4 CAR groups. **g** The heatmap of shared integrated genes in the D4 and HM2 CAR groups. The CAR T cells used in this figure were produced using the donor 1’s PBMCs. Values represent mean ± SEM. Source data and exact *p* values are provided in the Source data file.

To test the function of GPC1-targeted CAR T cells, we generated CARs using the singe-chain antibody variable fragment (scFv) of HM2 or the V_H_H nanobody D4 (Supplementary Table 4) and included the hinge (H) and transmembrane (TM) domains from CD8 along with a 4-1BB endodomain (Fig. 2a and Supplementary Fig. 5a). Among the four healthy donors-derived T cells used in our study, we noticed variations in the frequency of CD4^+^ (28–79.5%) and CD8^+^ (18.5–67%) T cells subpopulations (Supplementary Fig. 5b). The transduction efficiency of activated HM2 and D4 CAR T cells was 54% and 75%, respectively (Fig. 2b). To compare the cytolytic capacity of HM2 CAR and D4 CAR, CAR T cells were co-cultured with GPC1-negative A431 and GPC1-positive tumor cell lines including H8, 2B9 and T3M4 cells (Supplementary Fig. 5c–f). Both H8 and 2B9 cells were effectively lysed by HM2 and D4 CAR T cells even at low E:T ratios with similar potency. Minimal cell lysis was observed in A431 cells treated with GPC1-targeted CAR T cells, demonstrating target-dependent specificity. At the high E:T ratio of 30:1, HM2 CAR T cells and D4 CAR T cells killed 88% and 50% of T3M4 cells, which express low levels of GPC1. Although both HM2 and D4 CAR T cells exhibited similar killing ability, D4 CAR T cells triggered 2- to 7-fold more secretion of cytokines including IFN-γ, IL-2, and TNF-α than HM2 CAR T cells after exposure to GPC1-positive cancer cells (Fig. 2c and Supplementary Fig. 5g).

To evaluate the antitumor activities of GPC1-specific CAR T cells in vivo, NSG mice were intraperitoneally (i.p.) injected with 2B9-Luc-GFP cells. A single infusion of 10 million mock or CAR T cells was administered i.p. 11 days post inoculation (Fig. 2d). Both HM2 and D4 groups showed reduced tumor burden, while tumors continued to grow in the mock T-cell group (Fig. 2e, f). In addition, 40% and 60% of NSG mice that received HM2 and D4 CAR T cells, respectively, were alive without recurrence by week 4 post-infusion (Fig. 2f). We also assessed the persistence of CAR T cells using droplet digital PCR (ddPCR). In total, 13.9–35.7% of CAR vector-positive cells were found in the spleen of mice receiving HM2 and D4 CAR T cells at week 5 post-infusion (Supplementary Fig. 6a). Recent studies suggest that the precise location of CAR T vector integration into patients’ genome can play an essential role in the treatment outcome^26,27^. To investigate the integrated genes of GPC1-targeted CAR T-cell, we analyzed the lentiviral integration sites of HM2 and D4 CAR T cells recovered from the spleen and pancreas of mice. As shown in Fig. 2g and Supplementary Fig. 6b, HM2 CAR and D4 CAR showed an integration preference into distinct genes. In addition, the same integration sites were found in different tissues (e.g., spleen and pancreas) of the same mouse, indicating clonal expansion of CAR T cells in mice. Taken together, both HM2 and D4 CAR T cells persisted and regressed high GPC1-expressing xenograft tumors in mice.

### D4 CAR with a short IgG4 hinge regress low GPC1-expressing pancreatic cancer xenografts in mice

**Fig. 3:**
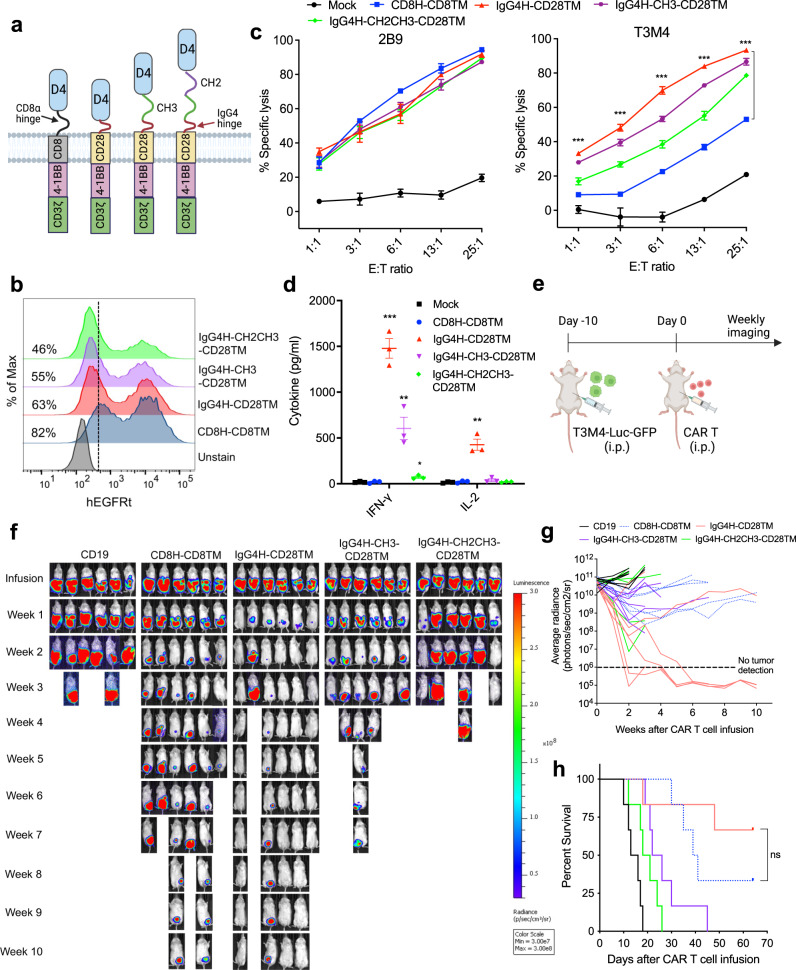
D4 CAR T cells with a shorter spacer domain significantly improve its reactivity against low GPC1-expressing tumor cells. **a** Schematics of D4-IgG4 hinge(H)-based CAR with different length of spacers (CH3, 103 aa and CH2CH3_,_ 216 aa). A shorter IgG4H (12 aa) was used to replace the original CD8H (45 aa). CD28 transmembrane (TM) domain was used in all D4-IgG4 hinge-based CARs. The illustration was created with BioRender.com. **b** Transduction efficiency of above D4 CAR constructs. Data are representative of two independent experiments. **c** The D4-IgG4H CAR T cells showed equally potent reactivity in high GPC1-expressing 2B9 cells, whereas demonstrated the best cytolytic activity when co-cultured with low GPC1-expressing T3M4 cells for 24 h. *n* = 3 independent experiments. ***p < 0.001, two-tailed unpaired Student’s *t* test. **d** Measurement of IFN-γ and IL-2 secretions in co-cultured supernatants with T3M4 cells at E:T ratio of 6:1. *n* = 3 independent experiments. *p = 0.016, **p < 0.01, ***p = 0.00017, two-tailed unpaired Student’s *t* test. **e** Experimental schematic (created with BioRender.com). T3M4 tumor-bearing NSG mice were treated with i.p. injection of 10 million mock T cells, D4-CD8H-CD8TM CAR T cells, D4-IgG4H-CD28TM CAR T cells, D4-IgG4H-CH3-CD28TM CAR T cells, and D4-IgG4H-CH2CH3-CD28TM CAR T cells on day 10 after tumor cell inoculation. *n* = 6 mice/group. **f** D4-IgG4H-CD28TM CAR T cells rapidly eliminated T3M4 tumor cells in mice, the ones with intermediate and long spacers only controlled tumor growth. **g** Tumor bioluminescence as photons per second in mice treated in (**f**). **h** Kaplan–Meier survival curve reveals a significant extended survival of mice receiving D4-IgG4H-CD28TM CAR T cells. ns, *p* = 0.2597, log-rank test. The CAR T cells used in this figure were produced using the donor 2’s PBMCs. Values represent mean ± SEM. Source data and exact *p* values are provided in the Source data file.

As our GPC1-targeted CARs showed modest antitumor activity against low GPC1-expressing T3M4 cancer cells (Supplementary Fig. 5f), there is a need to improve their potency. The D4 V_H_H-based CAR can be easily expressed and has a nearly 40% greater transduction efficiency than the HM2 scFv-based CAR (Fig. 2b). The D4 CAR T cells also induced more cytokine secretions upon stimulation (Fig. 2c) and resulted in tumor regression in 60% of mice compared with 40% tumor clearance in the HM2 CAR T-cell treatment group (Fig. 2f, g). Because of these benefits, we decided to further engineer the GPC1 CAR based on the D4 nanobody. A previous study demonstrated that the optimal spacer length of a given CAR depends on the position of the targeted epitope^9^. Since D4 recognizes a N-lobe epitope of GPC1, we hypothesized that shortening the spacer domain might improve T-cell signaling. Therefore, we replaced a 45 amino acid CD8H with a short 12 amino acid modified IgG4H (Supplementary Table 5 and Fig. 3a). To fully examine the impact of spacer length on CAR T-cell activation, we constructed two additional D4 CARs in which the modified IgG4-Fc spacer domain was sequentially added to derive D4-IgG4H-CH3 (medium) and D4-IgG4H-CH2CH3 (long) (Fig. 3a). All three D4-IgG4H-based CARs contain the CD28 TM domain. The expression of each of the CARs was confirmed (Fig. 3b). As shown in Fig. 3c, T cells expressing any of the D4-IgG4H-based CARs and the original D4-CD8H-CD8TM CAR killed high GPC1-expressing 2B9 cells equally well. All three D4-IgG4H-based CAR T cells showed improved reactivity compared to the D4-CD8H-CD8TM CAR T cells against low GPC1-expressing T3M4 cells. Notably, D4-IgG4H only CAR T cells demonstrated the highest cytotoxic potential. The potency is inversely correlated with the spacer length, further confirming that a short hinge benefits CARs targeted to membrane-distal epitopes. As observed in the cytolytic assay, the short spacer construct was superior in mediating IFN-γ and IL-2 secretions after recognition against T3M4 cells (Fig. 3d).

We then compared the antitumor activity of CAR T cells with different spacer lengths using the T3M4 i.p. xenograft mouse model (Fig. 3e). Mice treated with 10 million T cells expressing D4 CAR with IgG4H had rapid and complete tumor regression within 2 weeks of treatment (Fig. 3f, g). The same dose of D4 CAR T cells expressing either the medium or the long spacer was less effective in eliminating cancer cells in mice. D4-IgG4H only CAR T cells improved the survival of mice bearing T3M4 xenografts (Fig. 3h). Together, D4-IgG4H-CD28TM CAR T cells demonstrate enhanced antitumor efficacy in pancreatic cancer cells with low GPC1 antigen density.

### Both IgG4H and CD28TM regions contribute to the enhanced D4 CAR reactivity against low GPC1-expressing cancer cells

**Fig. 4:**
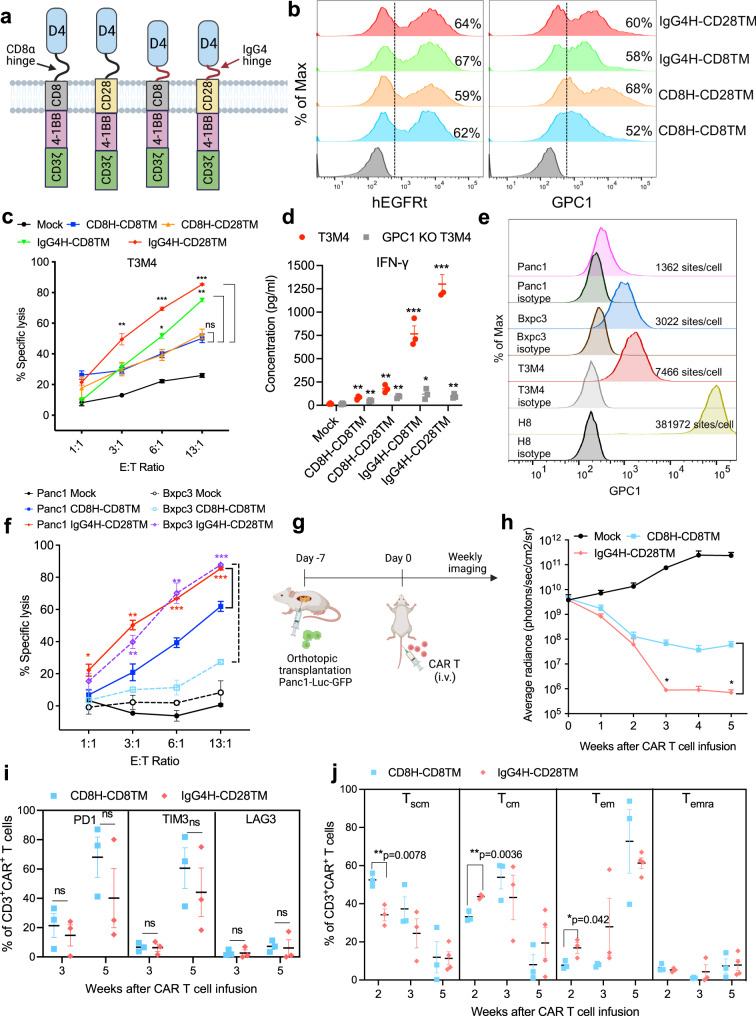
D4-IgG4H-CD28TM CAR T cells exhibit robust effector functions in vitro and in vivo. **a** Schematics of D4 CAR constructs differed in H and TM domains. The illustration was created with BioRender.com. **b** Transduction efficiency of four D4 CAR constructs as measured by hEGFRt expression and their binding to antigen GPC1. Data are representative of two independent experiments. **c** D4-IgG4H-CD28TM CAR T cells showed the best cytolytic activity among all four D4 CAR T cells when co-cultured with low GPC1-expressing T3M4 cells, but not GPC1 KO T3M4 cells. *n* = 3 independent experiments. ns = *p* > 0.05, *p = 0.022, **p < 0.01, ***p < 0.001, two-tailed unpaired Student’s *t* test. **d** D4-IgG4H-CD28TM CAR T cells induced the most secretion of IFN-γ upon stimulation with T3M4 cells at E:T ratio of 6:1. *n* = 3 independent experiments. *p = 0.032, **p < 0.01, ***p < 0.001, two-tailed unpaired Student’s *t* test. The CAR T cells used in (**b**–**d**) were produced using the donor 2’s PBMCs. **e** Cell surface expression of GPC1 on indicated pancreatic cancer cell lines. GPC1 molecules per cell as determined by QuantiBRITE phycoerythrin (PE) assay. *n* = 1 independent experiment. **f** D4-IgG4H-CD28TM CAR T cells potently lysed Bxpc3 (dotted lines) and Panc-1 (solid lines) cells after 24 h of co-culture. *n* = 3 independent experiments. *p = 0.034, **p < 0.01, ***p < 0.001, two-tailed unpaired Student’s *t* test. **g** Experiment schematic (created with BioRender.com). NSG mice were surgically implanted with Panc-1 cells in pancreas and treated with 5 million mock, D4-CD8H-CD8TM CAR, and D4-IgG4H-CD28TM CAR T cells on day 7 via tail vein injection. *n* = 5 mice/group. **h** Mice receiving D4-IgG4H-CD28TM CAR T cells were 100% tumor-free, whereas D4-CD8H-CD8TM CAR T group still carried small tumors. *n* = 5 mice/group. *p < 0.05, two-tailed unpaired Student’s *t* test. **i** Analysis of T-cell exhaustion in each group at different time points post-infusion. *n* = 3 mice/group. ns = *p* > 0.05, two-tailed unpaired Student’s *t* test. **j** Memory T-cell phenotypes of D4-CD8H-CD8TM and D4-IgG4H-CD28TM CAR T cells retrieved from mouse blood. *n* = 3 mice/group. Two-tailed unpaired Student’s *t* test. Relative proportion of stem cell-like memory (T_scm_ CD62L^+^CD45RA^+^CD95^+^), central memory (T_cm_ CD62L^+^CD45RA^−^CD95^+^), effector memory (T_em_ CD62L^−^CD45RA^−^CD95^+^), and terminally differentiated effector memory (T_emra_ CD62L^−^CD45RA^+^CD95^+^) phenotypes were defined by CD62L, CD45RA and CD95 expression. The CAR T cells used in (**f**–**j**) were produced using the donor 3’s PBMCs. Values represent mean ± SEM. Source data and exact *p* values are provided in the Source data file.

To investigate the role of IgG4H and CD28TM domains in the significantly improved potency of D4-IgG4H-CD28TM CAR, we generated two additional D4 CARs, D4-CD8H-CD28TM and D4-IgG4H-CD8TM (Fig. 4a). Similar expression of all four constructs were confirmed by staining with the anti-EGFR antibody cetuximab; however, their ability to bind antigen varied (Fig. 4b). T cells expressing D4-CD8H-CD8TM CAR showed the least binding to GPC1, and substitution of CD8TM to CD28TM increased the binding strength to GPC1. Replacing CD8H with IgG4H also improved the binding of D4 CAR to GPC1. Next, we examined the effect of H and TM on tonic signaling during ex vivo expansion. Replacing CD8TM with CD28TM in D4-CD8H CAR T cells increased the expression of T-cell activation (CD25) and exhaustion markers (e.g., PD1) (Supplementary Fig. 7a–c). This elevated tonic signaling of D4-CD8H-CD28TM CAR T cells was reduced by switching CD8H with IgG4H. We also analyzed the T differentiation subsets consisting of stem cell-like memory T cells (T_scm_: CD62L^+^CD45RA^+^CD95^+^), central memory T cells (T_cm_: CD62L^+^CD45RA^−^CD95^+^), effector memory T cells (T_em_: CD62L^−^CD45RA^−^CD95^+^) and terminally differentiated effector memory T cells (T_emra_: CD62L^−^CD45RA^+^CD95^+^). All three engineered D4 CARs increased the frequencies of T_em_ in CD4^+^ T-cell subpopulation and T_emra_ in CD8^+^ T-cell subpopulation compared with the original D4-CD8H-CD8TM CAR after ex vivo production (Supplementary Fig. 7d).

The D4-IgG4H-based CAR T cells showed significantly increased cytolytic activity against T3M4 cells compared with the D4-CD8H-based CAR T cells (Fig. 4c). The cytolytic activity of D4-IgG4H-CD28 TM CAR T cells is ~10% higher than D4-IgG4H-CD8TM CAR T cells against T3M4 cells. Substitution of CD8TM with CD28TM alone did not improve cell killing ability. Moreover, none of the four D4 CAR T cells lysed GPC1-knockout (KO) T3M4 cells (Supplementary Fig. 8), demonstrating target-dependent specificity. Consistent with cell killing potency, D4-IgG4H-CD28TM CAR T cells induced the most secretion of IFN-γ, IL-2, TNF-α, IL-17A, CXCL10, IL-4, IL-6, IL-8 and IL-10 upon stimulation with T3M4 cells (Fig. 4d and Supplementary Fig. 9). No difference was seen in the secretion of IL-12, TGF-β1 (free active), IL-1β and CCL-2 among different D4 CAR T cells.

We then measured the reactivity of D4-IgG4H-CD28TM CAR against other pancreatic cancer cell lines. Similar to T3M4 cells (7466 GPC1 sites/cell), Bxpc3 (3022 GPC1 sites/cell) and Panc-1 (1362 GPC1 sites/cell) cells also expressed low levels of GPC1 (Fig. 4e). The improved cytolytic activity of D4-IgG4H-CD28TM CAR T cells was also seen in Panc-1 and Bxpc3 cells compared with D4-CD8H-CD8TM CAR T cells (Fig. 4f). We further examined the antitumor activity of both D4 CAR T cells in an orthotopic pancreatic cancer mouse model. Panc-1-Luc-GFP cells were surgically implanted into mouse pancreas (Fig. 4g). After an initial reduction of tumor burden in both groups, D4-IgG4H-CD28TM T cells resulted in greater tumor regression than D4-CD8H-CD8TM CAR T cells (Fig. 4h and Supplementary Fig. 10a). At the end of this study, 100% of mice in the D4-IgG4H-CD28TM group remained tumor-free, whereas mice in the D4-CD8H-CD8TM group still carried small tumors (Supplementary Fig. 10a, b). During the study period, we analyzed CAR T cells from mouse peripheral blood at week 2, week 3 and week 5 (Supplementary Fig. 10c). An insignificantly lower number of circulating CAR T cells was found in the D4-IgG4H-CD28TM group compared to the counterpart D4-CD8H-CD8TM group (Supplementary Fig. 10d). The D4-IgG4H-CD28TM CAR T cells showed a slight increase of CD8^+^ subpopulation compared with the D4-CD8H-CD8TM CAR T cells at week 2, though this difference was not observed at week 5 (Supplementary Fig. 10e). In addition, the D4-IgG4H-CD28TM CAR T cells expressed lower levels of PD1 and TIM3 and stimulated more cytokine release than the D4-CD8H-CD8TM CAR T cells in mouse blood (Fig. 4i and Supplementary Fig. 10f). Moreover, CAR T cells recovered from mouse blood in the D4-IgG4H-CD28TM group were more differentiated than the D4-CD8H-CD8TM group by week 3 as evidenced by the increased proportion of T_em_ (Fig. 4j), which appear to be critical for profound antitumor activity. At week 5, an appreciably higher level of T_cm_ was found in circulating D4-IgG4H-CD28TM CAR T cells, likely contributing to the durable response. Taken together, we demonstrate that D4-IgG4H-CD28TM CAR T cells are persistent and able to drive the regression of multiple low-GPC1-expressing pancreatic tumors.

**Fig. 5:**
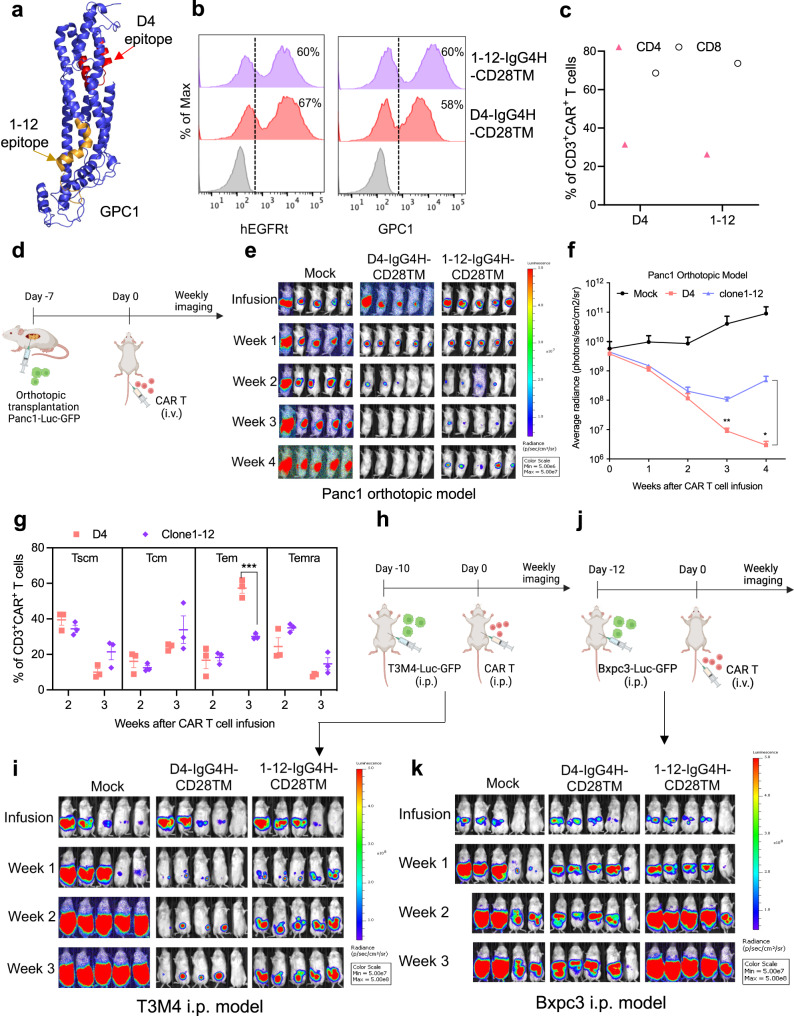
D4 and clone 1–12 CAR T cells have comparable antitumor activity in the IgG4H-CD28TM construct. **a** The epitopes of D4 (highlighted in red color) and clone 1–12 (highlighted in orange color) are found on the opposite sides of GPC1. **b** Transduction efficiencies of D4 and clone 1–12 CAR constructs as measured by hEGFRt expression and their binding to antigen GPC1. *n* = 1 independent experiment. **c** The CD4 and CD8 composition of pre-infusion D4-IgG4H-CD28TM and clone 1–12-IgG4H-CD28TM CAR T cells. *n* = 1 independent experiment. **d** Experiment schematic (created with BioRender.com). NSG mice were surgically implanted with Panc1 cells in pancreas and treated with 5 million mock, D4-IgG4H-CD28TM CAR, and clone 1–12-IgG4H-CD28TM CAR T cells on day 7 via tail vein injection. *n* = 5 mice/group. **e** Both D4 and clone 1–12 IgG4H-CD28TM CAR T cells induced tumor regression. **f** Tumor bioluminescence as photons per second in mice treated in (**e**). *n* = 5 mice/group. *p = 0.022, **p = 0.0018, two-tailed unpaired Student’s *t* test. Mice in the D4-IgG4H-CD28TM group showed lower tumor burden than its counterpart. **g** Memory T-cell phenotypes of D4-CD8H-CD8TM and clone 1–12-IgG4H-CD28TM CAR T cells retrieved from mouse blood. *n* = 3 mice/group. ***p = 0.0008, two-tailed unpaired Student’s *t* test. **h** Experimental schematic (created with BioRender.com). T3M4 tumor-bearing NSG mice were treated with i.p. injection of 10 million mock T cells, D4-IgG4H-CD28TM CAR T cells, and clone 1–12-IgG4H-CD28TM CAR T cells on day 10 after tumor cell inoculation. *n* = 5 mice/group. **i** D4-IgG4H-CD28TM CAR T cells were more effective in controlling T3M4 xenograft growth than clone 1–12-IgG4H-CD28TM CAR T cells. **j** Experimental schematic (created with BioRender.com). Bxpc3 tumor-bearing NSG mice were treated with i.v. injection of 10 million mock T cells, D4-IgG4H-CD28TM CAR T cells, and clone 1–12-IgG4H-CD28TM CAR T cells on day 12 after tumor cell inoculation. *n* = 5 mice/group. **k** D4-IgG4H-CD28TM CAR T cells demonstrated better antitumor activity than clone 1–12-IgG4H-CD28TM CAR T cells in the Bxpc3 i.p. mouse model. The CAR T cells used in this figure were produced using the donor 4’s PBMCs. Values represent mean ± SEM. Source data are provided as a Source Data file.

A prior study reported the potent antitumor effects of GPC1 CAR T cells utilizing the anti-GPC1 antibody clone 1–12^17^. The previously described epitope of clone 1–12^15^ is found on the opposite side of the D4 epitope and is located close to the cell membrane (Fig. 5a). The expression of D4-IgG4H-CD28TM and clone 1–12-IgG4H-CD28TM CARs were confirmed by both hEGFRt expression and GPC1 binding (Fig. 5b). A small decrease of D4 CAR expression as measured by GPC1 binding (58%) was found in comparison with hEGFRt expression (67%). The clone 1–12 CAR showed the same levels of expression (60%) by examining either marker. The pre-infusion clone 1–12-IgG4H-CD28TM CAR T cells appeared to have a slightly higher percentage of CD8^+^ T cells compared with the D4-IgG4H-CD28TM CAR T cells (Fig. 5c). Next, we compared the potency of D4 to that of clone 1–12 in the IgG4H-CD28TM CAR construct against low-GPC1-expressing pancreatic cancer cells. Both CAR T groups demonstrated similar cytolytic activity against Panc-1 cancer cells (Supplementary Fig. 11a). An improvement in killing potency against Bxpc3 and T3M4 cancer cells was observed in the D4-IgG4H-CD28TM CAR group compared with the clone 1–12-IgG4H-CD28TM group in culture. We then evaluated the efficacy of both CARs using the Panc-1 orthotopic pancreatic cancer mouse model (Fig. 5d). Both D4-IgG4H-CD28TM and clone 1–12-IgG4H-CD28TM CAR T cells induced tumor regression with comparable efficacy by week 2 (Fig. 5e, f). Notably, D4-IgG4H-CD28TM CAR T cells resulted in 100% complete tumor remission by week 4. The two groups exhibited indistinguishable amount of CAR T cells in animal peripheral blood (Supplementary Fig. 11b). At week 2, a slightly lower percentage of CD8^+^ CAR T cells in the clone 1–12 group was found in comparison with the D4 counterpart in mouse blood (Supplementary Fig. 11c). Consistent with our findings in Fig. 4j, circulating D4-IgG4H-CD28TM CAR T cells again favored a T_em_ phenotype at week 3 (Fig. 5g). In addition, D4-IgG4H-CD28TM CAR T cells were more effective in controlling tumor burden than clone 1–12-IgG4H-CD28TM CAR T cells in the T3M4 i.p. xenograft mouse model (Fig. 5h, i). Lastly, this greater antitumor efficacy of D4-IgG4H-CD28TM CAR T cells was also observed in the Bxpc3 i.p. xenograft mouse model (Fig. 5j, k). Overall, we demonstrate that D4 CAR T cells have comparable or potentially more potent antitumor efficacy than clone 1–12 CAR T cells in the IgG4H-CD28TM CAR format.

### D4-IgG4H-CD28TM CAR T cells are polyfunctional

Recent studies demonstrated that polyfunctional T cells (co-secretion of 2 or more cytokines/chemokines) determined at the single-cell level are the key effector cells contributing to the long-term immune response against various diseases^28–30^. The polyfunctional strength index (PSI) was calculated based on the percentage of polyfunctional cells and the intensities of secreted proteins. Only D4-IgG4H-CD28TM CAR T cells showed polyfunctionality in both CD4^+^ and CD8^+^ T-cell subpopulations and demonstrated a significant increase of PSI when stimulated with T3M4 cells in comparison to GPC1 KO T3M4 cells (Supplementary Fig. 12).

**Fig. 6:**
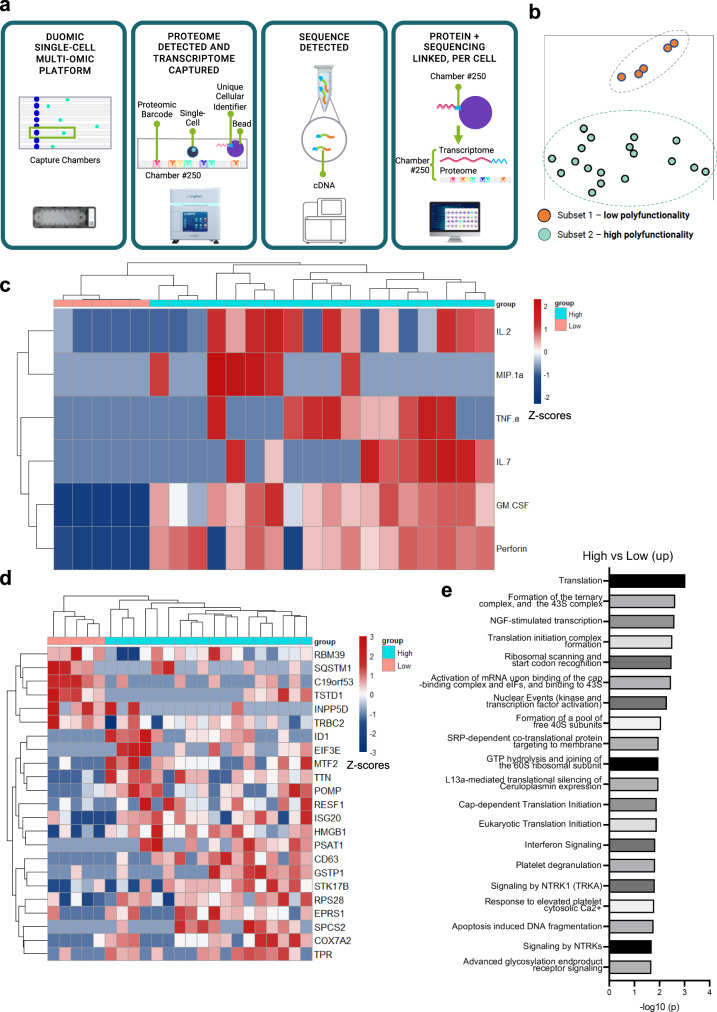
Single-cell analysis of polyfunctional CD8^+^ D4-IgG4-CD28TM CAR T cells at the mRNA and protein levels. **a** Flowchart of the single-cell protein and RNA expression analysis platform. The illustration was partially created with BioRender.com. **b** Identification of two CAR T clusters, low polyfunctionality and high polyfunctionality, in 23 CD8^+^ D4-IgG4H-CD28TM CAR T cells. **c** Functional analysis of two distinct CAR T clusters. **d** The corresponding RNA sequencing data reveals unique gene expression profiles for these two CAR-T clusters. **e** REACTOME pathway analysis of unique genes in (**c**). The CAR T cells used in this figure were produced using the donor 2’s PBMCs. Source data are provided as a Source Data file.

To understand the molecular determinants of D4-IgG4H-CD28TM polyfunctionality, we simultaneously measured the protein and RNA expression levels of 23 single CD8^+^ D4-IgG4H-CD28TM CAR T cells stimulated with T3M4 cells (Fig. 6a). We identified two CAR T clusters, low polyfunctionality and high polyfunctionality subsets, in a 2D t-SNE plot (Fig. 6b). The cytokines that were significantly upregulated in the high polyfunctionality CAR T subset include IL-2, IL-7, TNF-α, GM-CSF, perforin, and MIP-1α, all of which were expressed at low levels in the low polyfunctionality subset (Fig. 6c). Particularly, IL-2 and IL-7 are known to be important for T-cell survival and memory T-cell maintenance. Moreover, our single-cell mRNA (scRNA) expression profiling revealed 23 genes that displayed statistically significant, concordant differences between the two cell subsets (Fig. 6d and Supplementary Table 6). The upregulated genes in the high polyfunctional cell cluster play roles in promoting cell proliferation (e.g., *ID1*), cytokine signaling (e.g., *ISG20*), and immune responses (e.g., *HMGB1*), all of which are potentially associated with CAR T-cell efficacy. In contrast, upregulated genes (e.g., *INPP5D*) in the low polyfunctional cell cluster may have a negative impact on immune responses or cytokine signaling. A REACTOME pathway enrichment analysis was performed to further analyze two CAR T clusters. The 23 differentially expressed genes between two CAR T clusters in 44 correlated pathways were identified (Supplementary Table 7). The high polyfunctionality CAR T group contained genes associated with translation, formation of the ternary and 43S complex, NGF stimulated transcription, and translation initiation complex formation (Fig. 6e).

### D4-IgG4H-CD28TM CAR dimerizes to enhance T-cell function

**Fig. 7:**
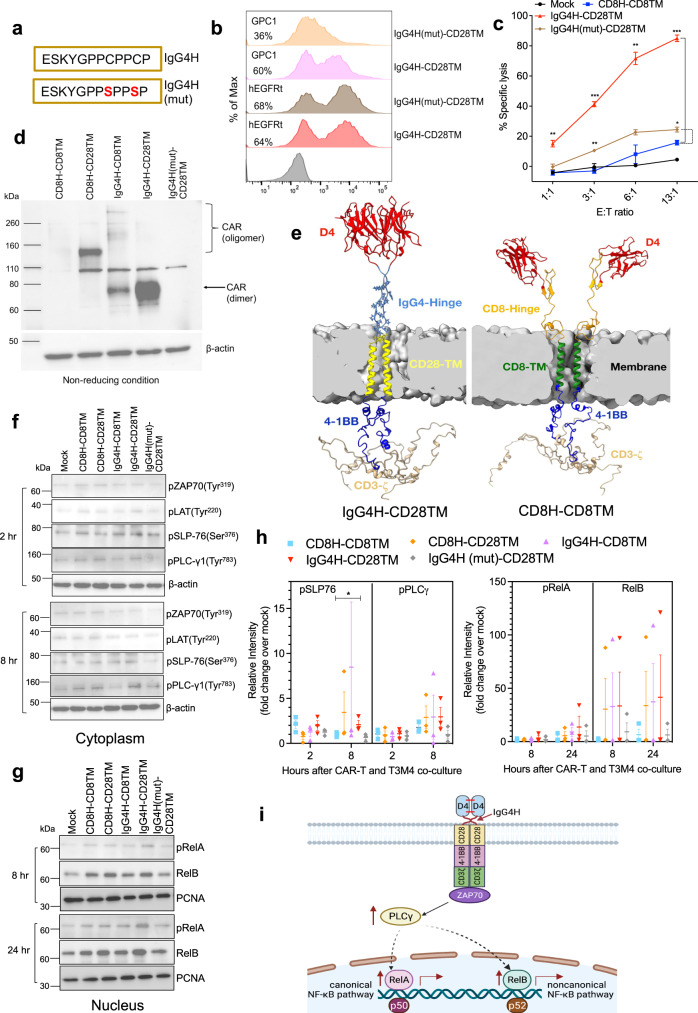
Dimerization of D4 CAR leads to the enhanced T-cell function. **a** Mutations of two cysteine residues in the hinge of D4-IgG4H-CD28TM CAR. **b** D4-IgG4H(mut)-CD28TM CAR T cells showed reduced binding to GPC1 though still had decent transduction efficiency determined by hEGFRt expression. Data are representative of two independent experiments. **c** D4-IgG4H-CD28TM CAR T cells lost the enhanced reactivity when both cysteine residues are mutated. *n* = 3 independent experiments. *p = 0.019, **p < 0.01, ***p < 0.001, two-tailed unpaired Student’s *t* test. **d** Representa*t*ive western blot of CAR expression under non-reducing condition. Membranes were stained with CD3ζ antibody. Experiments were repeated with similar results. **e** Modeling of D4-CD8H-CD8TM and D4-IgG4H-CD28TM CARs. **f** Western blot analysis of T-cell signaling molecules of various D4 CAR T cells upon stimulation with T3M4 cells in the cytoplasm. **g** Nuclear localization of transcription factors upon stimulate with T3M4 cells in the nucleus. **h** Blots in (**f**) and (**g**) were representative of three experiments using T cells prepared from three different donors. The compiled data were quantified using ImageJ and normalized with mock control. *p = 0.041, one-tailed unpaired Student’s *t* test. **i** Proposed mechanism of action (created with BioRender.com). D4-IgG4H-CD28TM CAR T cells are dimerized to enhance the phosphorylation of PLCγ in the cytoplasm and subsequently promote canonical and non-canonical NF-κB signaling, leading to the promising antitumor efficacy in low GPC1-expressing solid tumors. The CAR T cells used in this figure were produced using the donor 2’s and donor 4’s PBMCs. Values represent mean ± SEM. Source data and exact *p* values are provided in the Source data file.

We noticed the existence of two cysteine residues in the IgG4H that may form disulfide dimers to enhance T-cell signaling. To test the hypothesis, we performed cysteine-to-serine mutations (mut) in the IgG4H (Fig. 7a). Although D4-IgG4H(mut)-CD28TM CAR was properly expressed on T cells, mutations of the two cysteine residues led to a significant loss of binding to GPC1 (Fig. 7b). Furthermore, the enhanced cytolytic activity and cytokine (IFN-γ and IL-2) secretion of D4-IgG4H-CD28TM CAR T cells were lost when both cysteine residues were mutated (Fig. 7c and Supplementary Fig. 13a), indicating the interchain disulfide formation is crucial for D4-IgG4H CAR. Additionally, minimal cell lysis was observed in GPC1 KO-T3M4 cells (Supplementary Fig. 13b).

To further investigate the contribution of IgG4H and CD28TM to the dimerization of D4 CAR T cells, we performed western blot under reducing and non-reducing conditions. We found that IgG4H-based CARs, particularly with CD28TM, formed dimers with and without antigen stimulation (Fig. 7d and Supplementary Fig. 14), suggesting both IgG4H and CD28TM contribute to CAR dimerization. An oligomer was detected in the CD8H-CD28TM CAR molecule, whereas no dimer or oligomer was observed in CD8H-CD8TM nor IgG4H(mut)-CD28TM molecules. Predictive protein structural modeling of CD8H-CD8TM and IgG4H-CD28TM CAR molecules demonstrated the presence of D4 dimerization in IgG4H-CD28TM-containing CAR (Fig. 7e). The short (12 amino acid) IgG4H may induce a rigid structure to allow D4 intermolecular variable chain pairing to dimerize the ectodomain of D4-IgG4H-CD28TM CAR. The IgG4 hinge might also protrude closer onto the tumor antigen than the CD8 hinge. Our structure models also reveal that the involvement of IgG4H and CD28TM also makes intracellular 4-1BB and CD3ζ endodomains more organized and structured, which may lead to enhanced T-cell signaling via optimal binding and activation of downstream molecules. However, the involvement of CD8H and CD8TM causes the 4-1BB and CD3ζ endodomain less structured and poorly organized, which may lead to poor T-cell signaling. We co-cultured various D4 CAR T cells with T3M4 cells at different time points to test this hypothesis. CAR stimulation resulted in similar levels of phosphorylation of ZAP70 Ser^319^ and LAT Tyr^220^ in the cytoplasm (Fig. 7f). During a 2–8 h period, the phosphorylation of phospholipase Cγ (PLCγ) was mostly increased in IgG4H-CD28TM CAR T cells. Furthermore, we determined the nuclear translocation of transcription factors in T cells at later time points (after 8- and 24-h co-culture). All the engineered D4 CARs, particularly D4-IgG4H-CD28TM, enhanced the activation of both canonical nuclear factor κB (NF-κB) signaling (reflected by phospho-RelA/p65) and non-canonical NF-κB signaling (reflected by RelB) (Fig. 7g). We repeated the co-culture assay twice, quantified each protein band intensity and compared to the un-transduced mock control (Supplementary Fig. 15 and Fig. 7h). Our findings suggest that D4-IgG4H-CD28TM CAR T cells induce a steady phosphorylation of PLCγ upon antigen stimulation, which in turn activates NF-κB signaling (Fig. 7i). Taken together, D4-IgG4H-CD28TM CAR T cells are dimerized to promote T-cell signaling and enhance T-cell persistence.

**Fig. 8:**
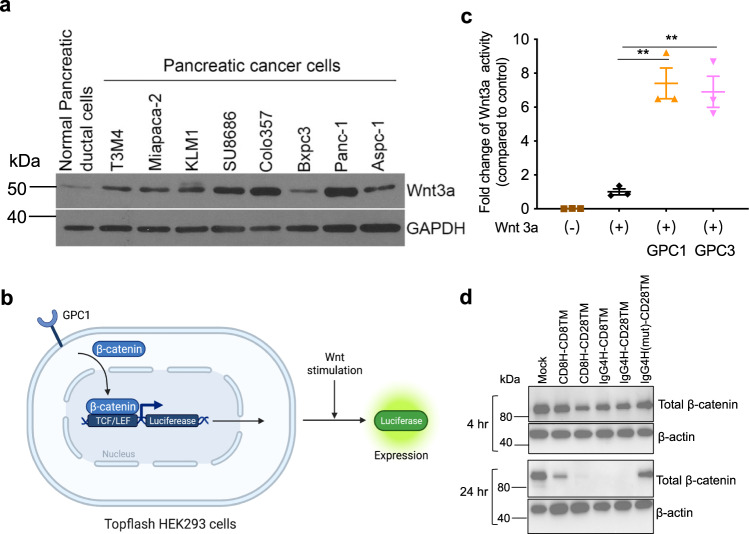
GPC1 is involved in Wnt signaling in pancreatic cancer. **a** Western blot analysis of Wnt3a expression in pancreatic cancer cell lines as well as the normal pancreatic duct cell line hTERT-HPNE. **b** Schematic of the TCF/LEF reporter assay (created with BioRender.com). **c** Overexpression of GPC1 increased TCF/LEF report activity. *n* = 3 independent experiments. **p < 0.01, two-tailed unpaired Student’s *t* test. **d** D4 CAR T cells inhibited Wnt signaling in T3M4 cells. Values represent mean ± SEM. Source data and exact *p* values are provided in the Source data file.

Glypicans (e.g., GPC2 and GPC3) have been reported to be extracellular co-receptors for Wnt proteins^31,32^. To evaluate the involvement of GPC1 in Wnt signaling in pancreatic cancer cells, we determined the expression of Wnt3a. Nearly all the pancreatic cancer cell lines except Bxpc3 showed increased Wnt3a protein compared to normal pancreatic duct cells (Fig. 8a). We then overexpressed GPC1 in HEK293 SuperTopflash cells, containing a luciferase reporter under the control of three LEF/TCF binding sites (Fig. 8b). Upon stimulation with Wnt3a conditioned medium, we found that overexpression of GPC1 significantly induced Wnt activation to the same extent as GPC3 overexpression (Fig. 8c). Moreover, all four D4 CAR T cells dramatically downregulated β-catenin expression in T3M4 cells, with the exception of IgG4H(mut)-CD28TM CAR likely due to its impaired binding ability to GPC1 (Fig. 8d), indicating that GPC1 CAR T cells may downregulate the Wnt/β-catenin signaling in target pancreatic tumor cells.

## Discussion

Preventing tumor escape due to low antigen expression remains a critical aspect of achieving clinical responses with CAR T cells in solid tumors, emphasizing a need to tune CAR function against low antigen density tumors. Here, we measured GPC1 antigen density on pancreatic cancer cell lines and observed low levels of GPC1 in pancreatic cancer. We developed a V_H_H nanobody (D4)-based CAR targeting a membrane-distal epitope of GPC1. By utilizing a rigid IgG4H and a CD28TM domain, D4 CAR significantly improved its activity against low GPC1-expressing xenograft tumors. We found that both IgG4H and CD28TM mediated D4 CAR dimerization to enhance T-cell signaling. D4-IgG4H-CD28TM CAR T cells are polyfunctional and exhibit a molecular signature supporting T-cell proliferation and long-term persistence. This work provides evidence for optimizing the recognition of targets and improving the function of nanobody-based CAR T cells in solid tumors.

Pancreatic cancer is characterized by the dense fibrotic stroma compartment that occupies most of the tumor mass. The roles of cancer-associated fibroblasts (CAFs) in pancreatic cancer have received more attention in recent years. We found that cell surface GPC1 expression is not only increased in pancreatic tumor tissues but also strongly elevated in CAFs compared with normal pancreas. A number of reports demonstrated that re-shaping the pancreatic stroma can impair tumor progression and improve therapeutic efficacy^33,34^. Therefore, further investigation of GPC1 expression in different CAF subtypes is warranted as well as whether our GPC1-specific CAR T cells could re-model the tumor microenvironment and destroy GPC1-positive pancreatic cancer cells. In addition to its membrane-anchored form, GPC1 also has a secreted soluble form and can be detected in the peripheral blood. Previous studies identified soluble GPC1 in pancreatic tumor cell-derived exosomes^35,36^. Thus, the potential resistance to GPC1 CAR T cells by soluble GPC1 needs to be validated.

The unique properties of V_H_H nanobodies include small size, high stability, simple engineering into multi-domain antibodies, and possible cross-reactivity in different species. The cross-reactivity of D4 to both human and murine GPC1 offers a tool to evaluate on-target off-tumor toxicities. The expression of mouse GPC1 is detected in several organs including brain, heart, testis, and others according to the mouse Gene Expression Database^37^. In our preclinical studies, D4-IgG4H-CD28TM CAR T cells exhibited potent antitumor activity in mice without any obvious adverse effects, indicating the safety of targeting GPC1 with D4 CAR T cells. To fully understand the binding patterns of the D4 antibody in tumor and healthy tissues, it would be important to engineer a tagged D4 recombinant protein that is suitable for immunostaining in future studies.

Recent studies suggest that the precise location of CAR T vector integration into patients’ genome can play an essential role in the treatment outcome^26,27,38^. We previously reported the integration sites of GPC3 and GPC2-targeted CAR in both responders and non-responders from preclinical mouse models^39,40^. In the current study, we analyzed the integration sites of GPC1-targeted CARs, aiming to identify a group of integrated genes commonly enriched in responders. Some of the genes, for example *ITGAL* (Integrin Subunit Alpha L), which is involved in leukocyte adhesion and transmigration, was also found in our GPC3 and GPC2-targeted CARs. These integrated genes enriched in responders are frequently associated with cell signaling and chromatin modification pathways, suggesting insertion in these genes may enhance T-cell activity under selection pressure. Further studies by increasing sample size in single-cell based analysis may lead to the discovery of cellular factors related to durable response to T-cell therapy.

The efficacy of D4-based CAR is greatly improved by IgG4H-CD28TM. In the Panc-1 orthotopic mouse model, the D4-IgG4H-CD28TM group remained tumor-free at the end of the study, whereas the D4-CD8H-CD8TM group carried small tumors. At week 3, we observed 3.5-fold more T_em_ in D4-IgG4H-CD28TM CAR T cells compared with D4-CD8H-CD8TM CAR T cells. T_em_ exhibits the highest level of cytotoxicity of all the memory cell subtypes^41^, which may explain the profound antitumor effect of D4-IgG4H-CD28TM CAR T cells in mice. By week 5, there was a 2.4-fold increase in T_cm_ in D4-IgG4H-CD28TM CAR T cells. T_cm_ phenotype has been correlated with sustained remissions in patients with leukemia^42^. Together, the induction of a robust T_cm_ population and a reduction of T-cell exhaustion in D4-IgG4H-CD28TM CAR T cells contribute to the potent and durable response in mice. Future studies using patient-derived xenograft models and primary tumor materials with heterogenous GPC1 expression will further validate the efficacy of D4-IgG4H-CD28TM CAR T cells in pancreatic cancer.

Our results demonstrated that both IgG4H and CD28TM are involved in the D4 CAR dimerization. Consistent with previous reports^43^, we confirmed that IgG4H formed disulfide bonds, but not for the IgG4H with mutations of two cysteine residues. The short IgG4H also connected two D4 antigen-binding domains, which was seen in our modeling of D4 CAR molecules and led to increased binding to GPC1. Our observation was also found in a recent study reporting that a connection of antigen-binding domains can drive 4-1BB-based CAR dimerization and antigen-independent signaling to enhance CAR function^44^. The CD28TM domain also mediated D4-IgG4H-CD28TM CAR dimerization, which directly impacts enhanced effector function in cell and mouse models. Although D4-IgG4H-CD28TM CAR forms dimers without antigen stimulation, we did not notice the increase of tonic signaling as measured by T-cell activation and exhaustion. Additionally, the CD28TM domain only facilitated oligomerization of D4-CD8H-CD28TM CAR, which resulted in increased tonic signaling and no improvement in CAR T activity compared with the CD8H-CD8TM CAR construct. Moreover, CAR dimerization is a structurally and functionally dynamic process. The dimerization of the D4-IgG4H-CD28TM CAR might be induced by the presence of the tumor antigen. The enhanced signaling of CAR T cells with the NF-κB signaling is found in the presence of the tumor antigen. Overall, our findings indicate dimerization could benefit a 4-1BB-based CAR function, although further investigation of CAR molecules targeting other antigens is needed.

We profiled the polyfunctionality of the CD8^+^ D4-IgG4H-CD28TM CAR T cells by analyzing single-cell protein and RNA expression from the same cells. We identified 23 genes that showed statistically significant differences between the high polyfunctionality and low polyfunctionality subsets. The upregulated genes in the high polyfunctional cell cluster play roles in promoting cell proliferation (e.g., *ID1*), cytokine signaling (e.g., *ISG20*), and immune responses (e.g., *HMGB1*), all of which are potentially associated with CAR T-cell efficacy. Furthermore, as an example of matched protein secretion and scRNA analysis, we observed an increased secretion of TNF-α in the high polyfunctionality CAR T cluster. Consistently, we noticed the upregulated expression of *HMGB1* gene in the same CAR T cluster. *HMGB1* is known to play an important role in the TNF-α-mediated inflammation signaling pathway^45^. Lastly, we acknowledge that the number of cells that can be analyzed in our current assay is limited and that future optimization of the methodology is needed to increase the single-cell output to reveal comprehensive molecular determinants of CAR T-cell polyfunctionality.

In summary, this work highlights a profound impact of antigen density on the potency of CAR T cells. By optimizing the hinge and transmembrane domains of the nanobody (D4)-based CAR, we have a potent lead GPC1 CAR T candidate that rapidly eliminates low GPC1-expressing xenografts which provides the opportunity for clinical applications in GPC1-positive cancers. We also illustrate different approaches to engineering a nanobody-based CAR recognizing a membrane-distal epitope of a tumor antigen. Our work therefore represents only the beginning for the study of nanobody-based CAR that clearly deserves further exploration.

## Methods

### Use of mice and use of human specimens in experiments

All experiments involving animals were approved by the Institutional Animal Care and Use Committee at the NIH (protocol LMB-059). Mice were housed under a standard 12:12 h light/dark cycle with a standard food and water.

Human peripheral blood samples from healthy donors were provided by the NIH Blood Bank (approved by the NIH Clinical Center Department of Transfusion Medicine). Informed written consent was obtained from all healthy donors for the use of their blood samples for laboratory research purposes by NIH researchers. Pancreatic tumor tissue microarray was purchased from US Biomax (Rockville, USA). The use of deidentified human specimens was determined to be the NIH Institutional Review Board (IRB) exempt. Because the specimens or data were not collected specifically for our study and no one on our study team has access to the subject identifiers linked to the specimens or data, our study is not considered as human subjects research.

### Isolation of anti-GPC1 antibodies

The isolation of mouse mAbs against glypican was described previously^46^. Hybridoma cells were screened via ELISA and flow cytometry. The HM2 clone demonstrating the highest affinity and most specific binding was chosen for purification. The D4 antibody was isolated from a large phage-displayed dromedary camel (*Camelus dromedarius*) V_H_H single-domain antibody library constructed in our laboratory using the polymerase chain reaction (PCR)-Extension Assembly and Self-Ligation (EASeL) method described previously^47,48^. Through three sequential rounds of panning on ELISA plate (Thermo Fisher Scientific) coated with human GPC1 in phosphate-buffered saline (PBS), GPC1-specific phages were enriched. Single colonies were then picked and identified by performing phage ELISA.

### Cell culture

The A431 (originally from ATCC #CRL-1555) and HEK-293T (originally from ATCC #CRL-3216) cell lines were obtained from Dr. Ira Pastan at the NCI. H8, a transfected A431 cell line stably expressing human GPC1, was made in our lab. HEK293 SuperTopflash stable cell line was obtained from Dr. Jeremy Nathans at the Johns Hopkins University. The aforementioned cell lines were cultured in DMEM supplemented with 10% FBS, 1% L-glutamine, and 1% penicillin–streptomycin at 37 °C in a humidified atmosphere with 5% CO_2_. Pancreatic cancer cell lines including T3M4, Aspc1 (originally from ATCC #CRL-1682), Bxpc3 (originally from ATCC #CRL-1687), Colo357, and SU8686 (originally from ATCC #CRL-1837) were obtained from Dr. Udo Rudloff at the NCI. Miapaca-2 (originally from ATCC #CRL-1420) and Panc-1 (originally from ATCC #CRL-1469) pancreatic cancer cell lines were obtained from Dr. Perwez Hussain at the NCI. KLM1 pancreatic cancer cell line was obtained from Dr. Christine Alewine at the NCI. PBMCs were isolated from the blood of healthy donors by Ficoll (GE Healthcare) according to the manufacturer’s instructions. These cells were grown in RPMI-1640 medium supplemented with 10% FBS, 1% L-glutamine, and 1% penicillin–streptomycin at 37 °C in a humidified atmosphere with 5% CO_2_. The hTERT-HPNE cell line (originally from ATCC #CRL-4023) was also obtained from Dr. Perwez Hussain at the NCI and cultured according to the provider’s instructions. A431, H8, KLM1, 2B9, T3M4, Bxpc3, and Panc-1 cell lines were engineered to express luciferase (Luc) and GFP in our lab.

### ELISA

Mouse hybridoma supernatant containing 1 μg/ml of each mAb was incubated with plates coated with human GPC1 through GPC6 purchased from R&D Systems. Binding was detected with a goat anti-mouse IgG conjugated with horseradish peroxidase (HRP) (Jackson ImmunoResearch, Cat#115-036-071) at 1:5000 dilution. The D4 camel V_H_H nanobody at 1 μg/ml was incubated with human GPC1 through GPC6 and mouse GPC1 proteins. Binding was detected with an anti-FLAG HRP-conjugated antibody (Sigma-Aldrich, Cat#A8592) at 1:5000 dilution. For the sandwich ELISA, the plate was coated with HM2 mAb in PBS. Recombinant human GPC1-hFc protein at concentrations of 5 µg/ml and 1 µg/ml were then added to the plate. After three washes, 0.4 μg/ml and 2 μg/ml were added to the plate. The bound D4 was detected by adding the anti-FLAG HRP-conjugated antibody.

Fifty three peptides of GPC1 were synthesized by Genscript. Each peptide is 18 amino acids long and has 9 overlapped amino acids with adjacent peptide. In total, 5 μg/ml peptides in PBS were used to coat the plates overnight. In total, 1 μg/ml of HM2 or D4 was added to the assay wells and the binding was detected.

### Antibody binding assay

The binding kinetics of HM2 and D4 antibody was measured with Octet RED96 system (FortéBio). For HM2, His-tagged GPC1 protein was immobilized onto Ni-NTA biosensor, which was subsequently used in association and dissociation measurements for a time window of 600 s and 1800 s, respectively. For D4, His-tagged D4 antibody was used to load the Ni-NTA biosensor, and serial diluted antigen human GPC1-hFc protein was used for binding assay. Data analysis was performed using the FortéBio analysis software.

### Immunohistochemistry

Pancreatic tumor tissue microarray was purchased from US Biomax. The sections were stained with 1 μg/ml HM2 mAb. The immunohistochemical staining was performed by Histoserv Inc.

### Reverse transcriptase-polymerase chain reaction (RT-PCR)

mRNA was isolated using a QuickPrep mRNA Purification kit (GE Healthcare), and first-strand cDNAs were synthesized using a SuperScript III First-Strand Synthesis System (Thermo Fisher Scientific) according to the manufacturer’s instructions. Primers designed to amplify GPC1 and β-actin are listed in Supplementary Table 8.

### CRISPR/Cas9-mediated editing of GPC1

The lentiCRISPRv2 expression vector was obtained from Dr. Feng Zhang (Addgene plasmid #52961). Two single-guide RNAs (sgRNAs) targeting the promoter region of GPC1 were cloned into the lentiCRISPRv2 vector following the protocol as described previously^49^. The sgRNAs sequences are listed in Supplementary Table 9. GPC1-knockout (KO)-T3M4 cells were obtained by single‐clone selection.

### Droplet digital PCR (ddPCR)

Tissues were homogenized using the Bullet Blender, and genomic DNA from cells was isolated using the FlexiGene DNA kit (QIAGEN). ddPCR experiments were performed on a QX200 ddPCR system (Bio-Rad) according to the manufacturer’s instructions. The primers and probes sequences were previously described^39^.

### Integration site analysis

CAR lentivector integration site analysis was performed using linker mediated PCR as described previously^50,51^. Briefly, sample DNA is randomly sheared, end-repaired, and ligated to a linker. The integration site is amplified with one primer specific to the lentivector LTR and another primer specific to the linker as previously described^39^. The amplified product is subjected to high-throughput Illumina Sequencing. Integration sites in the sample are identified and quantified for further analysis.

### Negative stain EM preparation and model building

The HM2 antigen-binding fragment (Fab) was prepared using a Fab preparation kit (Thermo Fisher Scientific). GPC1 protein was mixed with HM2 Fab at a 1:1 molar ratio in PBS. In addition, GPC1 protein was mixed with the D4-LR immunotoxin that contains domain III of PE at a 1:1 molar ratio in PBS. The details of data acquisition and 3D model construction were described previously^40^. Automatic model building was started with the production of RosettaFold, AlphaFold and YASARA models, and followed by the selection of best templates based on *Z*-score for all sequences, except for GPC1 in which PDB structures PDBID:4ACR, 4AD7, 4BWE and 4YWT were used as starting models. The models were placed in the maps using several criteria, including Segger (as implemented in Chimera), and the best poses were selected by hand. The models were further refined by Molecular Dynamics (MD) with CC constrains^52^. We modified the standard procedure to evaluate the ensemble evolution, instead of producing an average model. We extracted the highest probability model that fits the ensemble generated set when the ensemble average converges.

### Generation of GPC1-specific CAR T cells

The HM2 variable regions were cloned using 5’RACE with modified primers and conducted as described previously^53,54^. The antigen recognition regions from the HM2 (pMH304) or D4 (pMH305) antibody were subcloned into a lentiviral vector that contains expressing cassettes encoding the hinge and TM regions of CD8, a 4-1BB costimulatory domain, intracellular CD3ζ, the self-cleaving T2A sequence, and the truncated human epidermal growth factor receptor (hEGFRt) that was used as a surrogate marker for the CAR expression. A CD19-targeted CAR with the hinge and TM from CD8α was used as a control.

The CD8 hinge in the initial D4 CAR construct was replaced with a modified human IgG4 hinge (S$\to$P substitution)^11^ followed by either CD8TM (pMH382) or CD28TM (pMH377) domain. Another D4 CAR, D4-CD8H-CD28TM (pMH402), was also constructed. An additional spacer domain derived from a modified human IgG4-Fc previous studies^6,11^ was added into the D4-IgG4H-CD28TM CAR construct: D4-IgG4H-CH_3_-CD28TM CAR (pMH378) and D4-IgG4H-CH_2_CH_3_-CD28TM CAR (pMH379). Specifically, the first six amino acids (APEFLG) were replaced with five amino acids (APPVA), and a mutation (N297Q) at a glycosylation site in the CH_2_ domain. Previous reports demonstrated that these 4/2NQ modifications could prevent Fc receptor binding^6,7^. Additionally, two cysteine residues in the hinge of D4-IgG4H-CD28TM CAR were mutated to serine (pMH383, D4-IgG4H(mut)-CD28TM CAR). The anti-GPC1 mAb clone 1–12 scFv sequence obtained from US patent 10,077,316 B2 was synthesized and cloned into IgG4H-CD28TM CAR construct.

The CAR T cells were produced as described previously^32,39^. Briefly, PBMCs from healthy donors were stimulated for 24 h with anti-CD3/anti-CD28 antibody coated beads (Invitrogen) at a 2:1 bead-to-T-cell ratio in growth medium supplemented with IL-2. Activated bulk CD3^+^ T cells were then transduced with the lentivirus expressing GPC1-specific CARs at a multiplicity of infection (MOI) of 5. Cells were counted every other day and fed with fresh growth medium every 2–3 days. T cells were used for in vitro and in vivo studies after 7–9 days of expansion.

### In vitro functional assay

The cytolytic activity of GPC1-targeted CAR T cells were determined using a luciferase-based assay as previously described^32,39^. Briefly, GPC1-targeted CAR T cells and mock T cells were co-cultured with GPC1-positive pancreatic cancer cells (T3M4, Bxpc3, Panc-1), GPC1-overexpressing cells (2B9 derived from KLM1, H8 derived from A431), and GPC1-negative cells (GPC1-knockout-T3M4, A431) at different ratios for 24 h. All cancer cells were engineered to express luciferase (Luc) and GFP. The amount of CAR T cells used in each assay was normalized based on transduction efficiency. The luciferase activity was measured using the luciferase assay system (Promega) on SpectraMax Luminometer (Molecular Devices). In each assay, various GPC1-targeted CAR T cells were generated from the same PBMC donor for comparison.

### Cytokine assays

IFN-γ, TNF-α, and IL-2 secretion in the co-cultured supernatants were measured by using ELISA (R&D Systems) following manufacturer’s instructions. To comprehensively compare secretions of cytokines and chemokines by different D4 CAR T cells, a fluorescence-encoded bead-based multiplex assay (Biolegend) was performed according to the manufacturer’s protocol.

### Flow cytometry

T3M4 pancreatic cancer cells were incubated with mouse hybridoma supernatant containing 10 μg/ml of each mAb. Cell binding was then detected with a goat anti-mouse IgG conjugated with phycoerythrin (PE) (Jackson ImmunoResearch, Cat#115-116-072) at 1:200 dilution. For analysis of GPC1 expression on the cell surface, cancer cells were incubated with 10 μg/ml of HM2 and D4 and detected with a goat anti-mouse IgG conjugated with allophycocyanin (APC) (Jackson ImmunoResearch, Cat#115-136-072) at 1:200 dilution and an anti-FLAG antibody conjugated with APC (Biolegend, Cat#637308) at 1:100 dilution, respectively. To measure lentiviral transduction efficiencies, CAR expression on T cells was either detected with the anti-EGFR human monoclonal antibody cetuximab (Erbitux) at 1 μg/ml or GPC1-hFc protein produced in our lab at 5 μg/ml and goat anti-human IgG conjugated with PE (Jackson ImmunoResearch, Cat#109-116-097) at 1:200 dilution. In mouse experiments, peripheral CAR T cells were distinguished by staining for BV421 CD45 (Biolegend, Cat#368522); BV605 CD3 (Biolegend, Cat#317322); APC-H7 CD4 (BD Bioscience, Cat#560158); and PerCP-Cy5.5 CD8 (Biolegend, Cat#344710). CAR expression was detected with the anti-EGFR human monoclonal antibody cetuximab (Erbitux) at 1 μg/ml and goat-anti-human IgG conjugated with Alexa Fluor 488 (Jackson ImmunoResearch, Cat#109-546-097) at 1:200 dilution. Counting beads (123count eBeads, Thermo Fisher Scientific) were used to quantify absolute CAR T-cell number in mouse blood. T-cell exhaustion was assessed by staining for PE PD1 (Biolegend, Cat# 379210), PE-Cy7 TIM3 (Biolegend, Cat#345014) and APC LAG3 (Biolegend, Cat# 369212). In a separate panel, T-cell immunophenotyping was performed by surface staining with antibodies against the following antigens BV421 CD45RA (BD Bioscience, Cat# 562885), APC CD62L (BD Bioscience, Cat# 566791); and PE CD95 (Biolegend, Cat# 305608). T-cell activation was evaluated via PE CD25 (Thermo Fisher Scientific, Cat#12-0257-42); PE CD27 (Thermo Fisher Scientific, Cat#12-0279-42); and PE CD127 (Thermo Fisher Scientific, Cat#12-1278-42). All fluorochrome-conjugated primary antibodies were used at 5 μl/sample. FACS plots representing CAR T-cell data were conducted on gated CD3^+^CAR^+^ cells. Data acquisition was performed using SONY ID7000 (Sony Biotechnology) and analyzed using FlowJo software (Tree Star). The cytokines and chemokines were also analyzed using the Human Essential Immune Response or Human CD8/NK Panel (LEGENDplex, Biolegend) as per the manufacturer’s instructions. Analysis was performed by flow cytometry using LSR-Fortessa cytometer (Becton Dickinson), and data was processed using LEGENDplex Data Analysis Software (Biolegend).

### Single-cell cytokine profiling of T-cell polyfunctionality

Viable CAR T cells were enriched using Ficoll. CD4^+^/CD8^+^ T-cell subsets were separated using anti-CD4 or anti-CD8 microbeads (Miltenyi Biotec) and stimulated with T3M4 and GPC1 KO T3M4 cells at a ratio of 1:2 for 20 h. A single-cell functional profile was determined as described previously^28,30^. Each sample’s polyfunctionality strength index (PSI) was computed using a prespecified formula^28^, defined as the percentage of polyfunctional cells, multiplied by the mean fluorescence intensity (MFI) of the proteins secreted by those cells.

### Simultaneous measurements of protein and scRNA expression from the same single CAR T cells

CD8^+^ D4-IgG4H-CD28TM CAR T cells were co-cultured with T3M4 cells at a 1:2 E:T ratio for 24 h. CD8^+^ D4-IgG4H-CD28TM CAR T cells were then loaded onto the Duomic chip (IsoPlexis) for single-cell protein evaluation. A custom 18-plex antibody panel was used: Alpha Tubulin, GM-CSF, Granzyme B, INF-g, IL-10, IL-2, IL-7, IL-8, MIP-1a, MIP-1b, P-IkBA, P-MEK1-2, P-NK-kB p65, P-p44-42 MAPK, p-Stat 1, p-Stat 5, Perforin, TNF-a. Simultaneously, the mRNA molecules are captured onto the microbeads. Each chamber of a Duomic chip houses an mRNA capture bead with a unique molecular barcode sequence. A custom adapter and sequencing primer were used for read 1. Standard Illumina adapter (Illumina, FC-131-1001) and sequencing primer were used for read 2. Samples were sequenced on the Illumina NextSeq1000 instrument using the manufacturer’s protocol (Illumina, 20046811). Read lengths were as follows: 50 cycles read 1, 8 cycles index, 75 cycles read 2. RCutadapt version 3.4 was utilized to trim nucleotide bases from read 1 that were not relevant for cell barcode identification. Subsequently Alevin Salmon version 1.4.0 was utilized for cell barcode identification and gene expression quantification. KMeans clustering on data and RTsne v. 0.15 were utilized to perform the T-SNE dimension reductionality technique. KMeans labels calculated on the protein data were then utilized to apply the same cell labels for the gene expression data. Differential expression analysis was performed utilizing DESeq2 v 1.30.1 with genes with a nominal *p* value less than 0.05 considered to be significantly differentiated. Heatmaps were generated utilizing the R pheatmap library v. 1.0.12. Reactome Pathway analysis was performed utilizing Reactome Pathway Browser v 3.7.

### 3D molecular modeling of D4 CARs

CAR sequences were processed using RossettaFold, AlphaFold and I-Tasser^55–57^. The best initial partial models (as ranked by Z-core) were used as input to generate a more completed model using Yasara v20^58^ homology macro, and hand corrected to satisfy the requirements of the transmembrane domain and hinge dimer. The model^59,60^ was then inserted in a membrane using Yasara md_runmembrane with default parameters, restraining the model using the feedback restraint molecular dynamics protocol, followed by MD relaxation.

### CAR stimulation and protein lysate generation

Transduced T cells were serum starved overnight to reduce background phosphorylation. Different D4 CAR T cells normalized with transduction efficiency were incubated with T3M4 cancer cells at the E:T ratio of 5:1. After the allotted time, cells were quickly collected, washed with ice-cold PBS, and then lysed to collect both nuclear and cytoplasmic fractionations using the NE-PER nuclear and cytoplasmic extraction reagent kit (Thermo Fisher Scientific) per the manufacturer’s instructions. Protein concentration was quantified by bicinchoninic acid (BCA) protein assay (Thermo Fisher Scientific).

### Western blot

Equal masses of protein lysates were loaded onto a 4–20% SDS-PAGE gel for electrophoresis. HM2 was used to detect GPC1 expression at 1 μg/ml. An anti-CD3ζ antibody was purchased from Santa Cruz Biotechnology (Cat#sc-166435, 1:200 dilution). Antibodies including anti-ZAP70-pTyr319 (Cat#2701S, 1:500 dilution), anti-LAT-pTyr220 (Cat#3584S, 1:500 dilution), anti-SLP-76-pSer376 (Cat#14745S, 1:500 dilution), anti-PLC-γ1-pTyr783 (Cat#2821S, 1:500 dilution), anti-RelA/p65-pSer536 (Cat#3033S, 1:500 dilution), anti-RelB (Cat#10544S, 1:500 dilution), anti-β-catenin (Cat#9582S, 1:1000 dilution), anti-β-actin (Cat#8457S, 1:2000 dilution), GAPDH (Cat#5174S, 1:5000 dilution), and anti-PCNA (Cat#2586S, 1:1000 dilution) were obtained from Cell Signaling Technology. Anti-Wnt3a was purchased from Abcam (Cat#ab28472, 1:100 dilution). Band intensities were quantified using ImageJ (NIH) and normalized to a mock sample.

### Wnt reporter assay

HEK293 SuperTopflash cells were seeded into a 48-well plate. Cells were transfected with GPC1 or GPC3-expressing plasmid the next day. After 48 h of transfection, an equal volume of Wnt3a conditioned medium was added to cells. Luciferase activity was measured and then normalized with total protein after 6 h.

### Animal studies

Five-week-old female NOD/SCID/IL-2Rgc^null^ (NSG) mice (NCI CCR Animal Resource Program/NCI Biological Testing Branch) were housed and treated under the protocol (LMB-059) approved by the Institutional Animal Care and Use Committee at the NIH. For the 2B9 intraperitoneal (i.p.) model, 2 million Luc-GFP-expressing 2B9 (2B9-Luc-GFP) cancer cells were i.p. injected into mice. Mice with established tumors were then randomly allocated into three groups and i.p. injected once with 10 million T cells as follows: (1) un-transduced T cells (Mock); (2) HM2 CAR T cells; (3) D4 CAR T cells. For the T3M4 i.p. model, 2 million T3M4-Luc-GFP cancer cells were i.p. injected into mice. Mice with established tumors were randomly allocated into groups including mock and various formats of D4 CAR T cells with different hinge and TM domains. Mock or D4 CAR T cells were i.p. infused once at doses indicated in the figure legends. In a separate experiment, 10 million D4-IgG4H-CD28TM CAR or clone 1–12-IgG4H-CD28TM CAR T cells were i.p. infused once into mice. For the Bxpc3 i.p. model, 2 million Bxpc3-Luc-GFP cancer cells were i.p. injected into mice. Mice with established tumors were randomly allocated into three groups (mock, D4-CD8H-CD8TM, and D4-IgG4H-CD28TM) and i.v. infused once with 10 million T cells. To compare the efficacy of D4 and clone 1–12 in the IgG4H-CD28TM CAR format, 10 million CAR T cells were i.v. infused once into mice. For the Panc-1 orthotopic model, 0.25 million Panc-1-Luc-GFP cancer cells mixed with Matrigel at 1:1 volume ratio were surgically implanted into mouse pancreas. Mice with established tumors were randomly allocated into three groups (mock, D4-CD8H-CD8TM, and D4-IgG4H-CD28TM) and i.v. infused once with 5 million T cells. In another experiment, 5 million D4-IgG4H-CD28TM CAR or clone 1–12-IgG4H-CD28TM CAR T cells were i.v. infused once into mice bearing Panc-1 orthotopic pancreatic tumors. In each animal study, various GPC1-targeted CAR T cells were generated from the same PBMC donor. Four donors’ PBMCs were used throughout the manuscript. To detect the tumor growth and survival of mice, all mice were injected i.p. weekly with 3 mg D-luciferin (PerkinElmer) and imaged 10 min later using Xenogen IVIS Lumina (PerkinElmer). Living Image software was used to analyze the bioluminescence signal flux for each mouse as photons per second per square centimeter per steradian (photons/s/cm^2^/sr). Mice reached endpoint criteria and euthanized when any of the following conditions were observed: tumor interfered with animals’ ability to eat or drink, 20% weight loss, or any sign of outward distress such as hunched posture, ruffled fur, and reduced motility.

### Statistical analysis

Data analysis and visualization was performed using GraphPad Prism and are presented as mean ± SEM. Results were analyzed using two-tailed unpaired Student’s *t* test. Kaplan–Meier survival curve was analyzed using log-rank. A *p* value of <0.05 was considered statistically significant and and *p* values are denoted with asterisks as follows (***p < 0.001, **p < 0.01, *p < 0.05, ns = *p* > 0.05). Number of repeats performed is described in the relevant figure legend.

### Reporting summary

Further information on research design is available in the Nature Portfolio Reporting Summary linked to this article.

## Supplementary information

Supplementary Information

Reporting Summary

## Data Availability

Several GPC1 structures including 4ACR, 4AD7, 4BWE, and 4YWT in the PDB database were used as models in the study. Raw and processed data from the scRNA sequencing experiments are deposited and available in the NCBI’s Gene Expression Omnibus (GEO) database under accession code GSE220536. The remaining data are available within the article, Supplementary Information or Source Data files. Source data are provided with this paper.

## Source data

Source Data
