# Supplementary material for: The IgG4 hinge with CD28 transmembrane domain improves VHH-based CAR T cells targeting a membrane-distal epitope of GPC1 in pancreatic cancer
Source: Nat Commun. 2023 Apr 8;14:1986. doi: 10.1038/s41467-023-37616-4 (PMC10082787; doi:10.1038/s41467-023-37616-4)
Supplement: Supplementary file 1 — Supplementary Information [file 41467_2023_37616_MOESM1_ESM.pdf]

Supplementary Table 1: **Protein production yield of anti-GPC1 mouse monoclonal antibodies.**

**Antibody**	HM1	HM2	HM3	HM4	HM5	HM6
**Concentration (μg/ml)**	12.3	22.3	7.4	7.6	4.1	7.8

Supplementary Table 2: The amino acid sequence of the GPC1 peptides. Each peptide is 18 amino acids long and has 9 overlapped amino acids with adjacent peptides.

Name	Sequence	Name	Sequence
peptide 1	DPASKSRSCGEVRQIYGA	peptide 29	LKGCLANQADLDAEWRNL
peptide 2	GEVRQIYGAKGFSLSDVP	peptide 30	DLDAEWRNLLDSMVLITD
peptide 3	KGFSLSDVPQAEISGEHL	peptide 31	LDSMVLITDKFWGTSGVE
peptide 4	QAEISGEHLRICPQGYTC	peptide 32	KFWGTSGVESVIGSVHTW
peptide 5	RICPQGYTCCTSEMEENL	peptide 33	SVIGSVHTWLAEAINALQ
peptide 6	CTSEMEENLANRSHAELE	peptide 34	LAEAINALQDNRDTLTAK
peptide 7	ANRSHAELETALRDSSRV	peptide 35	DNRDTLTAKVIQGCGNPK
peptide 8	TALRDSSRVLQAMLATQL	peptide 36	VIQGCGNPKVNPQGPGPE
peptide 9	LQAMLATQLRSFDDHFQH	peptide 37	VNPQGPGPEEKRRRGKLA
peptide 10	RSFDDHFQHLLNDSERTL	peptide 38	EKRRRGKLAPRERPPSGT
peptide 11	LLNDSERTLQATFPGAFG	peptide 39	PRERPPSGTLEKLVSEAK
peptide 12	QATFPGAFGELYTQNARA	peptide 40	LEKLVSEAKAQLRDVQDF
peptide 13	ELYTQNARAFRDLYSELR	peptide 41	AQLRDVQDFWISLPGTLC
peptide 14	FRDLYSELRLYYRGANLH	peptide 42	WISLPGTLCSEKMALSTA
peptide 15	LYYRGANLHLEETLAEFW	peptide 43	SEKMALSTASDDRCWNGM
peptide 16	LEETLAEFWARLLERLFK	peptide 44	SDDRCWNGMARGRYLPEV
peptide 17	ARLLERLFKQLHPQLLLP	peptide 45	ARGRYLPEVMGDGLANQI
peptide 18	QLHPQLLLPDDYLDCLGK	peptide 46	MGDGLANQINNPEVEVDI
peptide 19	DDYLDCLGKQAEALRPFG	peptide 47	NNPEVEVDITKPDMTIRQ
peptide 20	QAEALRPFGEAPRELRLR	peptide 48	TKPDMTIRQQIMQLKIMT
peptide 21	EAPRELRLRATRAFVAAR	peptide 49	QIMQLKIMTNRLRSAYNG
peptide 22	ATRAFVAARSFVQGLGVA	peptide 50	NRLRSAYNGNDVDFQDAS
peptide 23	SFVQGLGVASDVVRKVAQ	peptide 51	NDVDFQDASDDGSGSGSG
peptide 24	SDVVRKVAQVPLGPECSR	peptide 52	DDGSGSGSGDGCLDDLCS
peptide 25	VPLGPECSRAVMKLVYCA	peptide 53	DGCLDDLCSRKVSRKSSS
peptide 26	AVMKLVYCAHCLGVPGAR	peptide 54	RKVSRKSSSRTPLTHAL
peptide 27	HCLGVPGARPCPDYCRNV	peptide 55	SRTPLTHALPGLSEQEGQ

peptide 28	PCPDYCRNVLKGCLANQA	peptide 56	PGLSEQEGQKTSAAS

Supplementary Table 3: The detailed information of each tissue specimen in Supplementary Figure 4.

Position	Pathology	Stage	TNM	Type
A1	Duct adenocarcinoma	I	T2N0M0	Malignant
A2	Duct adenocarcinoma	I	T2N0M0	Malignant
A3	Duct adenocarcinoma (sparse)	II	T3N0M0	Malignant
A4	Duct adenocarcinoma	III	T3N1M0	Malignant
A5	Duct adenocarcinoma	II	T3N0M0	Malignant
A6	Duct adenocarcinoma	I	T2N0M0	Malignant
A7	Duct adenocarcinoma	II	T3N0M0	Malignant
A8	Duct adenocarcinoma (sparse)	II	T3N0M0	Malignant
A9	Duct adenocarcinoma	IV	T3N0M1	Malignant
A10	Duct adenocarcinoma	III	T2N1M0	Malignant
B1	Duct adenocarcinoma	II	T3N0M0	Malignant
B2	Duct adenocarcinoma	II	T3N0M0	Malignant
B3	Duct adenocarcinoma	II	T3N0M0	Malignant
B4	Duct adenocarcinoma	II	T3N0M0	Malignant
B5	Duct adenocarcinoma	II	T3N0M0	Malignant
B6	Duct adenocarcinoma	I	T2N0M0	Malignant
B7	Duct adenocarcinoma (sparse)	II	T3N0M0	Malignant
B8	Duct adenocarcinoma	I	T2N0M0	Malignant
B9	Duct adenocarcinoma	II	T2N0M0	Malignant
B10	Duct adenocarcinoma	I	T2N0M0	Malignant
C1	Duct adenocarcinoma	II	T3N0M0	Malignant
C2	Duct adenocarcinoma	II	T3N0M0	Malignant
C3	Duct adenocarcinoma	II	T3N0M0	Malignant
C4	Duct adenocarcinoma (sparse)	II	T3N0M0	Malignant
C5	Duct adenocarcinoma	II	T3N0M0	Malignant
C6	Duct adenocarcinoma	II	T3N0M0	Malignant
C7	Duct adenocarcinoma	II	T3N0M0	Malignant
C8	Duct adenocarcinoma	II	T3N0M0	Malignant
C9	Duct adenocarcinoma (sparse)	III	T2N1M0	Malignant
C10	Duct adenocarcinoma	II	T3N0M0	Malignant
D1	Duct adenocarcinoma	III	T4N1M0	Malignant
D2	Duct adenocarcinoma	I	T2N0M0	Malignant
D3	Duct adenocarcinoma	I	T2N0M0	Malignant

D4	Duct adenocarcinoma (sparse)	II	T3N0M0	Malignant
D5	Duct adenocarcinoma	I	T2N0M0	Malignant
D6	Duct adenocarcinoma	I	T1N0M0	Malignant
D7	Duct adenocarcinoma	III	T3N1M0	Malignant
D8	Duct adenocarcinoma	III	T3N1M0	Malignant
D9	Duct adenocarcinoma	III	T2N1bM0	Malignant
D10	Duct adenocarcinoma	II	T3N0M0	Malignant
E1	Duct adenocarcinoma	III	T3N1M0	Malignant
E2	Duct adenocarcinoma	I	T2N0M0	Malignant
E3	Duct adenocarcinoma	I	T2N0M0	Malignant
E4	Duct adenocarcinoma	II	T3N0M0	Malignant
E5	Duct adenocarcinoma	II	T3N0M0	Malignant
E6	Duct adenocarcinoma	I	T2N0M0	Malignant
E7	Duct adenocarcinoma	I	T2N0M0	Malignant
E8	Duct adenocarcinoma	II	T3N0M0	Malignant
E9	Duct adenocarcinoma	I	T2N0M0	Malignant
E10	Duct adenocarcinoma	III	T2N1M0	Malignant
F1	Duct adenocarcinoma	II	T3N0M0	Malignant
F2	Duct adenocarcinoma (sparse)	I	T2N0M0	Malignant
F3	Duct adenocarcinoma	III	T2N1M0	Malignant
F4	Duct adenocarcinoma	II	T3N0M0	Malignant
F5	Duct adenocarcinoma	II	T3N0M0	Malignant
F6	Duct adenocarcinoma	IV	T3N0M1	Malignant
F7	Duct adenocarcinoma	II	T3N0M0	Malignant
F8	Duct adenocarcinoma	II	T3N0M0	Malignant
F9	Acinic cell carcinoma	I	T2N0M0	Malignant
F10	Squamous cell carcinoma	II	T3N0M0	Malignant
G1	Adjacent normal pancreas tissue	-	-	NAT
G2	Adjacent normal pancreas tissue	-	-	NAT
G3	Adjacent normal pancreas tissue	-	-	NAT
G4	Adjacent normal pancreas tissue	-	-	NAT
G5	Adjacent normal pancreas tissue	-	-	NAT
G6	Adjacent normal pancreas tissue	-	-	NAT
G7	Pancreas tissue	-	-	Normal
G8	Pancreas tissue	-	-	Normal

G9	Pancreas tissue	-	-	Normal
G10	Pancreas tissue	-	-	Normal

Supplementary Table 4: The amino acid sequences of HM2 scFv and D4 V_HH.

Name	Sequence	Patent Application
HM2 scFv	EVQLQQSGAELVRPGASVKLSCTASGFNIKDDYMHWVKQR PEQGLEWIGWIDPENGDTEYASKFQGKATITADTSSNTAYLQ LSSLTSEDTAVYYCTRSSVGYWGQGTTLTVSSGGGGSGGGG SGGGGSDVVMTQTPLSLPVSLGDQASISCRSSQSLVHSNGNT YLHWYLQKPGQSPKLLIYKVSNRFSGVPDRFSGSGSGTYFTL KISRVEAEDLGVYFCSQRTHVPYTFGGGTKLEIK	PCT/US2020/ 013739
D4 V_HH	QVQLVESGGGLVQPGGSLRLSCVASGYSYSIGYMAWFRQAP GKERAWVASRYTGDGGAVFDDAVKGRFTTSQESAGNTFDL QMDSLKPEDTAMYYCAAKGPGFGRWEYWGRGTQVTVSS	PCT/US2020/ 013739

Supplementary Table 5: Protein sequences of CD8 hinge and IgG4 hinge.

Hinge	Sequence
CD8	TTTPAPRPPTPAPTIASQPLSLRPEACRPAAGGAVHTRGLDFACD
Modified IgG4	ESKYGPPCPPCP

Supplementary Table 6: List of genes that are significantly enriched in low and high polyfunctionality subsets of CD8+ D4-IgG4H-CD28TM CAR T cells. The DESeq2, an R library, was used for the differential expression analysis test.

**Gene**	**Base mean**	**Log2 fold change**	**P value**	**Functional cluster**	**Major function**
GSTP1	0.883	-0.929	0.040	high	↓TNF-a; ↑anti-apoptosis
ID1	0.830	-0.933	0.036	high	↑cell growth
MTF2	0.977	-0.917	0.043	high	↑chromatin regulator, DNA-binding
CD63	0.953	-1.237	0.006	high	↑cell survival
EIF3E	0.809	-0.888	0.045	high	↑protein synthesis
HMGB1	2.576	-1.180	0.005	high	↑immune response
TPR	1.278	-1.036	0.022	high	↑cell cycle/division
COX7A2	0.991	-1.059	0.019	high	↑oxidative phosphorylation
PSAT1	0.792	-1.001	0.025	high	↑amino-acid biosynthesis
ISG20	1.356	-0.996	0.027	high	↑cytokine signaling in immune system
EPRS1	0.942	-0.934	0.039	high	↑protein synthesis and translation regulation
POMP	1.259	-0.919	0.042	high	↑proteasome assembly and maturation
RPS28	5.002	-0.858	0.009	high	↑ribosomal protein
SQSTM1	0.610	0.939	0.036	low	immune system process; Interleukin-1 signaling
RBM39	2.685	0.714	0.041	low	mRNA splicing major pathway
C19orf53	0.505	0.914	0.041	low	unknown
TRBC2	4.969	0.561	0.041	low	antigen and immunoglobulin binding
TSTD1	0.636	0.887	0.049	low	↑enables thiosulfate-thiol sulfurtransferase activity
INPP5DD	0.585	0.966	0.028	low	negative regulation of immune response

Supplementary Table 7: Reactome pathway analysis of genes that are significantly enriched in low and high polyfunctionality subsets of CD8^{+} D4-IgG4H-CD28TM CAR T cells. A hypergeometric distribution test, which is corrected for false discovery rate using the Benjamani-Hochberg method, was performed in this study.

Gene Pathway	P value	Genes
Translation	0.003	*SPCS2; RPS28; EPRS1; EIF3E*
Formation of the ternary complex, and subsequently, the 43S complex	0.004	*RPS28; EIF3E*
NGF-stimulated transcription	0.005	*ID1*
Translation initiation complex formation	0.006	*RPS28; EIF3E*
Ribosomal scanning and start codon recognition	0.006	*RPS28; EIF3E*
Activation of the mRNA upon binding of the cap-binding complex and eIFs, and subsequent binding to 43S	0.007	*RPS28; EIF3E*
Signaling by ALK fusions and activated point mutants	0.007	*TPR; SQSTM1*
Signaling by ALK in cancer	0.007	*TPR; SQSTM1*
Nuclear Events (kinase and transcription factor activation)	0.009	*ID1*
Cytokine Signaling in Immune system	0.012	*ISG20; INPP5D; TPR; HMGB1;SQSTM1*
Formation of a pool of free 40S subunits	0.016	*RPS28; EIF3E*
Interleukin-1 signaling	0.017	*HMGB1; SQSTM1*
SRP-dependent co-translational protein targeting to membrane	0.020	*SPCS2; RPS28*
GTP hydrolysis and joining of the 60S ribosomal subunit	0.020	*RPS28; EIF3E*
L13a-mediated translational silencing of Ceruloplasmin expression	0.020	*RPS28; EIF3E*

Cap-dependent Translation Initiation	0.023	*RPS28; EIF3E*
Eukaryotic Translation Initiation	0.023	*RPS28; EIF3E*
Pexophagy	0.023	*SQSTM1*
Apoptosis induced DNA fragmentation	0.023	*HMGB1*
PECAM1 interactions	0.025	*INPP5D*
NF-kB is activated and signals survival	0.027	*SQSTM1*
Platelet degranulation	0.027	*CD63; TTN*
Signaling by NTRK1 (TRKA)	0.028	*ID1*
Advanced glycosylation endproduct receptor signaling	0.029	*HMGB1*
p75NTR recruits signaling complexes	0.029	*SQSTM1*
Response to elevated platelet cytosolic Ca2+	0.030	*CD63; TTN*
Metabolism of amino acids and derivatives	0.031	*RPS28; TSTD1; EPRS1; PSAT1*
NRIF signals cell death from the nucleus	0.032	*SQSTM1*
p75NTR signals via NF-kB	0.034	*SQSTM1*
Interferon Signaling	0.034	*ISG20; TPR*
Signaling by NTRKs	0.037	*ID1*
Interleukin-1 family signaling	0.037	*HMGB1; SQSTM1*
PINK1-PRKN Mediated Mitophagy	0.039	*SQSTM1*
Sulfide oxidation to sulfate	0.039	*TSTD1*
Synthesis, secretion, and inactivation of Glucose-dependent Insulinotropic Polypeptide (GIP)	0.039	*SPCS2*
Influenza Viral RNA Transcription and Replication	0.041	*RPS28; TPR*
Immune System	0.042	*ISG20; CD63; TSTD1; GSTP1; INPP5D; TPR; HMGB1; SQSTM1*

Selenoamino acid metabolism	0.042	*RPS28; EPRS1*
tRNA processing	0.043	*EPRS1; TPR*
Serine biosynthesis	0.045	*PSAT1*
Interferon alpha/beta signaling	0.045	*ISG20*
MyD88 deficiency (TLR2/4)	0.046	*HMGB1*
Synthesis, secretion, and deacylation of Ghrelin	0.046	*SPCS2*
IRAK4 deficiency (TLR2/4)	0.048	*HMGB1*

Supplementary Table 8: Sequences of primers used for GPC1 and β-actin amplifications.

Name	Sequence (5' to 3')
Human GPC1-forward	GCAGCGTGCACACGTGGCTG
Human GPC1-reverse	CTGGCCCTTACAGTAGCCAGGC
Human β-actin-forward	CACCATTGGCAATGAGCGGTTC
Human β-actin-reverse	AGGTCTTTGCGGATGTCCACGT

Supplementary Table 9: Sequences of sgRNAs used for GPC1 KO generation.

sgRNA	Sequence (5'-3')
sgRNA 1	CCTCTCCCGCGGCCGCCTAG
sgRNA 2	GAGCGAGCGTTCGGACCTCG

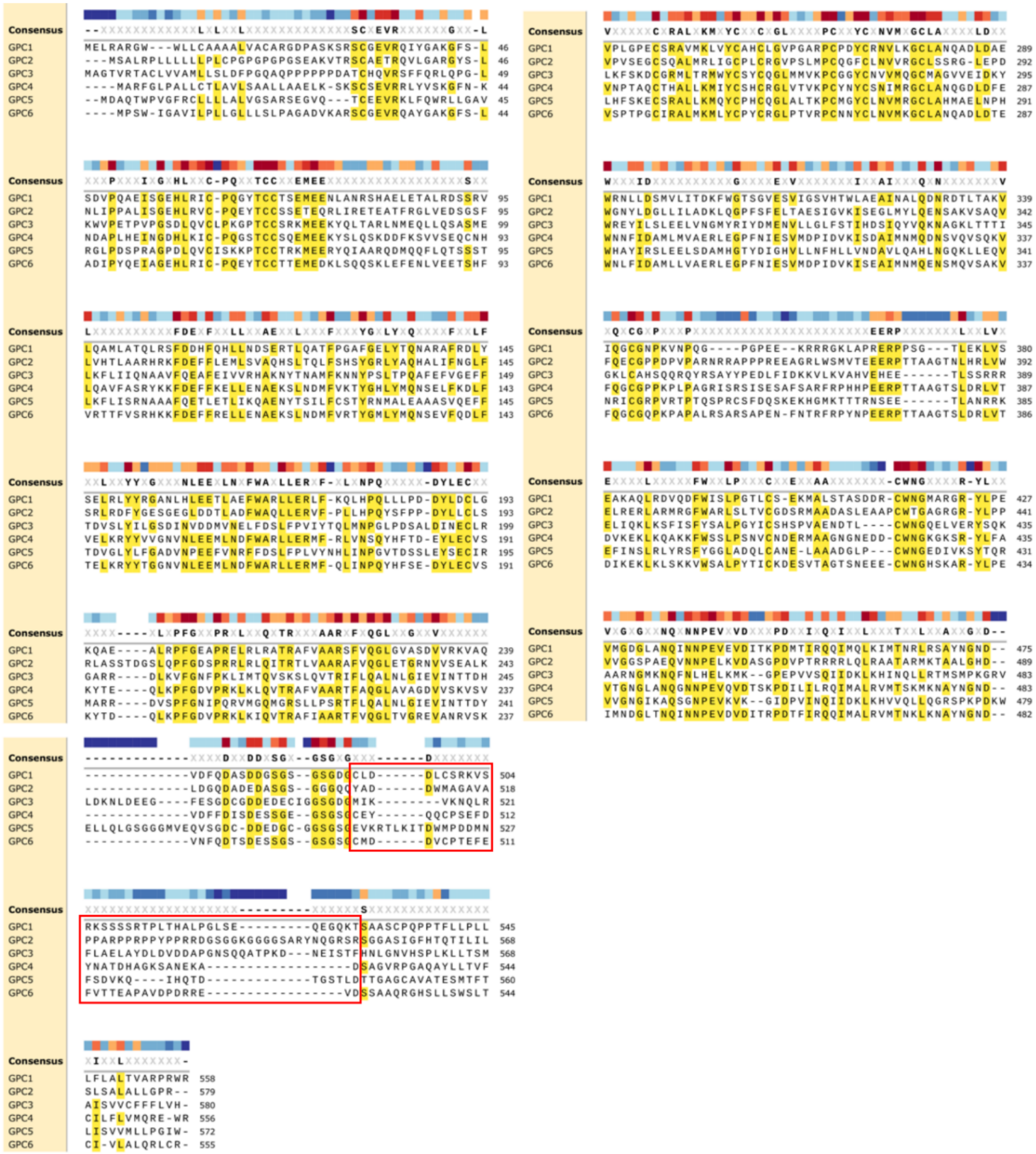

Supplementary Fig. 1: Alignment of human glypican amino acid sequences. The alignment was performed using MUSCLE. Amino acids that matched are marked with yellow highlighting. The C-terminal regions of glypicans are highlighted in red boxes.

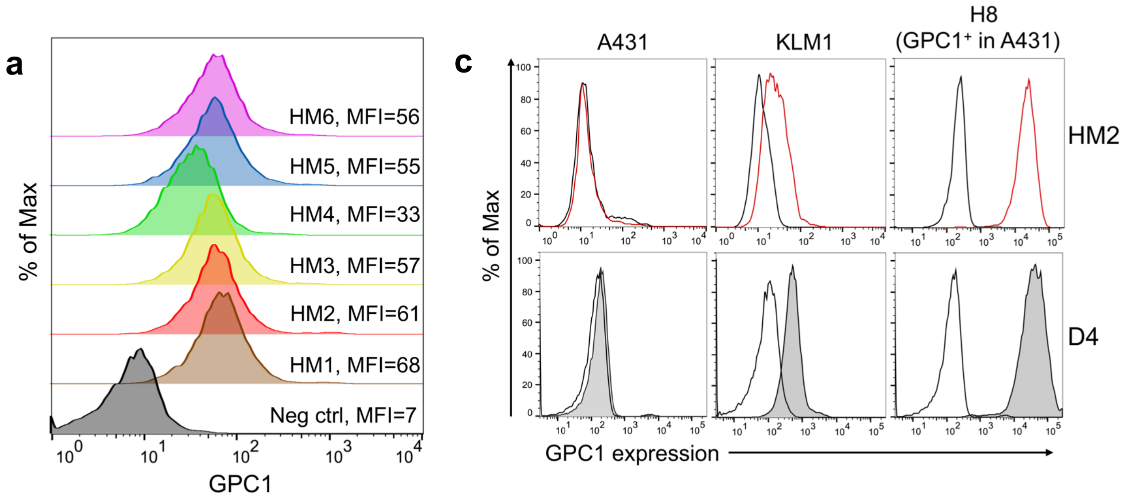

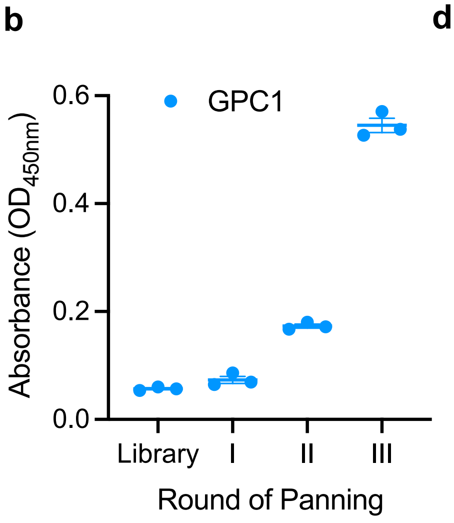

d
MELRARGWWLLCAAAALVACARGDPASKSRSCGEV
RQIYGAKGFSLSDVPQAEISGEHLRICPQGYTCCTSE
MEENLANRSHAELETALRDSSRVLQAMLATQLRSFD
DHFQHLLNDSERTLQATFPGAFGELYTQNARAFRDL
YSELRLYYRGANLHLEETLAEFWARLLERLFKQLHPQ
LLLPDDYLDCLGKQAEALRPFGEAPRELRLRATRAFV
AARSFVQGLGVASDVVRKVAQVPLGPECSRAVMKLV
YCAHCLGVPGARPCPDYCRNVLKGCLANQADLDAE
WRNLLDSMVLITDKFWGTSGVESVIGSVHTWLAEAI
NALQDNRDTLTAKVIQGCGNPKVNPQGPGPEEKRR
RGKLAPRERPPSGTLEKLVSEAKAQLRDVQDFWISL
PGTLCSEKMALSTASDDRCWNGMARGRYLPEVMG
DGLANQINNPEVEVDITKPDMTIRQQIMQLKIMTNRL
RSAYNGNDVDFQDASDDGSGSGSGDGCLDDLCSR
KVSRKSSSSRTPLTHALPGLSEQEGQKTSAASCPQP
PTFLLPLLLFLALTVARPRWR

Supplementary Fig. 2: Isolation of GPC1-specific antibodies and characterization of their binding epitopes. a, Flow cytometry comparing 6 mouse mAb at 10 µg/ml showed all increased binding to GPC1-positive T3M4 pancreatic cancer cells compared with non-specific control IgG. n = 1 independent experiment. **b**, GPC1 protein was used for panning on camel nanobody phage displayed library. Polyclonal phage ELISA from the output phage of each round of panning. n = 3 independent experiments. **c**, Binding abilities of HM2 and D4 to GPC1 expressed on cell surface. H8 is a GPC1-overexpressing A431 cell line. KLM1 is a pancreatic cancer cell line. **d**, GPC1 amino acid sequence and the epitopes of HM2 and D4. D4 reacted with two peptides including peptide 14 (blue colored) and peptide 15 (underlined). HM2 reacted with peptide 53 (red colored). Values represent mean ± SEM. Source data are provided as a Source Data file.

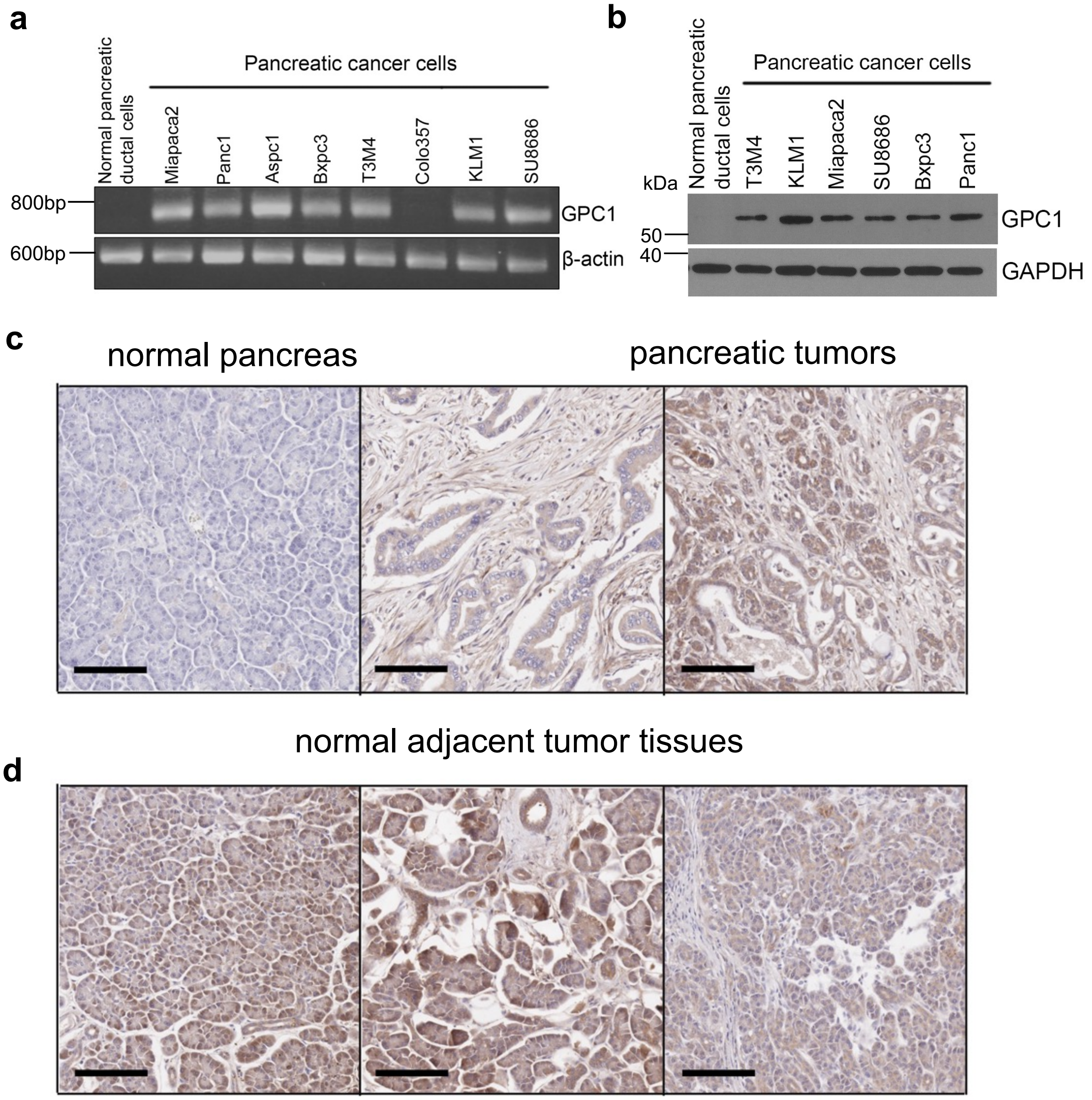

Supplementary Fig. 3: Increased expression of GPC1 in pancreatic cancer. a, GPC1 mRNA levels are increased in a majority of pancreatic cancer cell lines including Miapaca-2, Panc-1, Aspc-1, Bxpc3, T3M4, Colo357, KLM1 and SU8686 compared with normal pancreatic duct cell line hTERT-HPNE. The experiment was repeated twice with similar results. **b**, GPC1 protein levels are also elevated in pancreatic cancer cells compared with normal pancreatic duct cell line hTERT-HPNE. HM2 antibody was used to detect GPC1 protein in the western blot. The experiment was repeated twice with similar results. **c**, GPC1 expression is detected in pancreatic tumor tissues at various levels as compared to normal pancreas. These are representative images of Supplementary Fig. 4 including 50 specimens from patients of pancreatic cancer, 6 specimens

of tumor-adjacent normal tissues and 4 normal pancreatic tissues. **d**, GPC1 expression is detected in NATs. 1 μg/ml of HM2 was used for IHC. Scale bar = 100 μm. Source data are provided as a Source Data file.

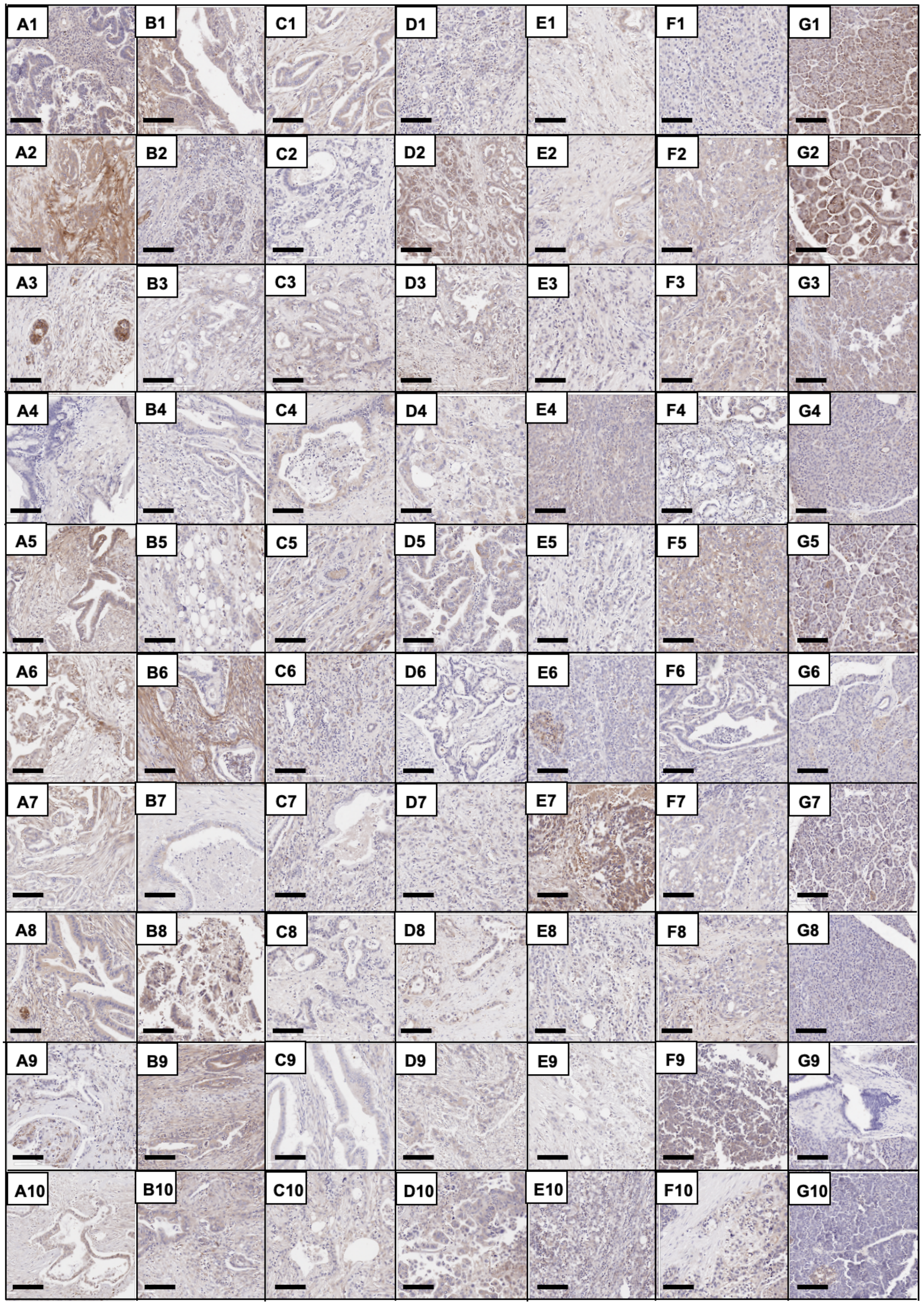
A1
B1
C1
D1
E1
F1
G1
A2
B2
C2
D2
E2
F2
G2
A3
B3
C3
D3
E3
F3
G3
A4
B4
C4
D4
E4
F4
G4
A5
B5
C5
D5
E5
F5
G5
A6
B6
C6
D6
E6
F6
G6
A7
B7
C7
D7
E7
F7
G7
A8
B8
C8
D8
E8
F8
G8
A9
B9
C9
D9
E9
F9
G9
A10
B10
C10
D10
E10
F10
G10

Supplementary Fig. 4: GPC1 expression as determined by immunohistochemistry. The staining was performed once. This tissue microarray includes 50 specimens from patients of pancreatic cancer, 6 specimens of tumor-adjacent normal tissues and 4 normal pancreatic tissues. The tissues were labeled with 1 μg/ml HM2 antibody. Images were obtained under 20X magnification. Scale bar = 100 μm.

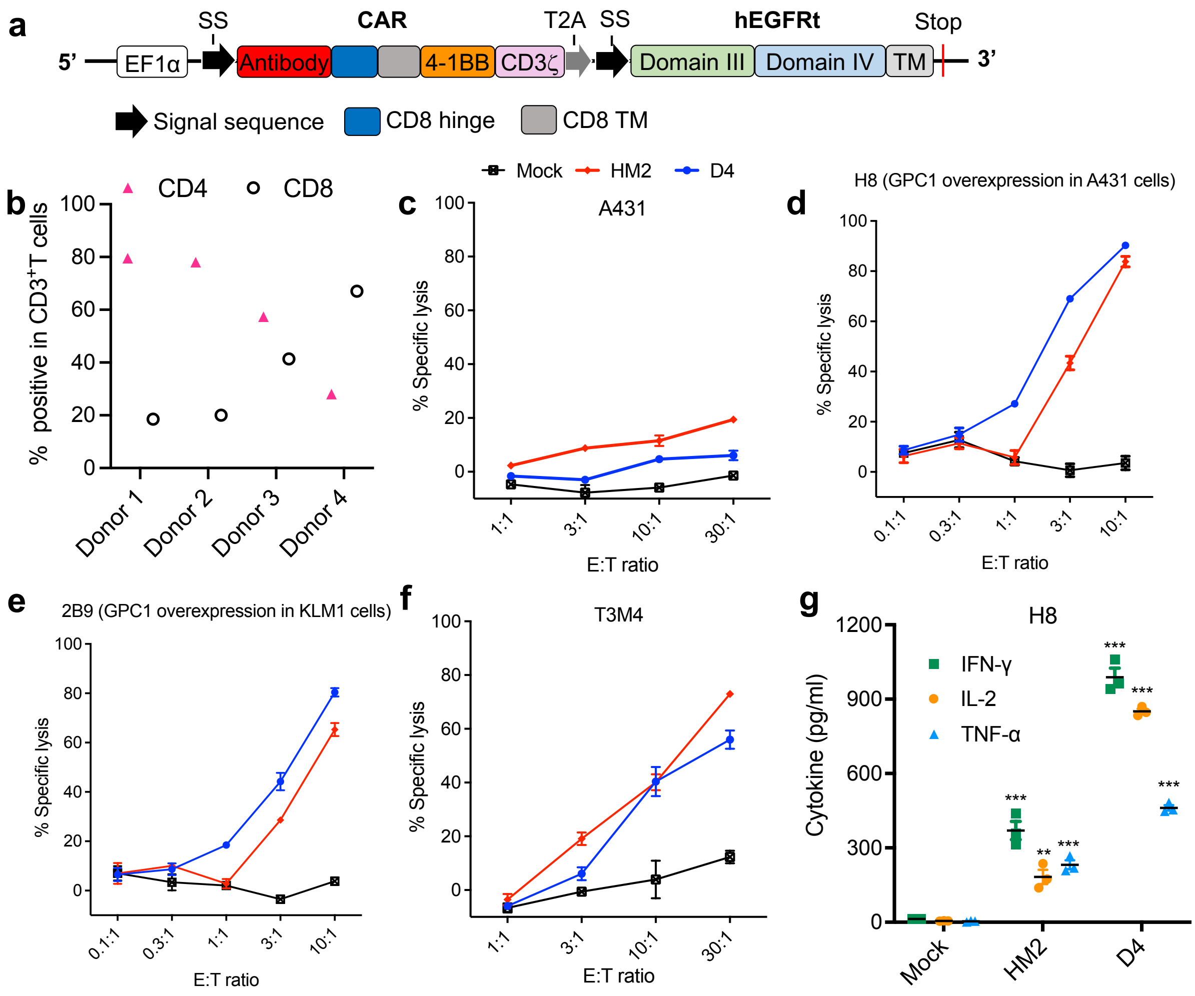

Supplementary Fig. 5: GPC1-targeted CAR T cells kill GPC1-positive tumor cells *in vitro*.
a, Schematic of the CAR construct. Co-expression of the truncated human epidermal growth factor receptor (hEGFRt) in this bicistronic vector is used for cell tracking and ablation. The hEGFRt lacks the domains essential for ligand binding and tyrosine kinase activity, but it retains the binding epitope of anti-EGFR monoclonal antibody cetuximab. **b,** The percentages of $CD4^+$ and $CD8^+$ T cells in T cells derived from four healthy donors. **c-f**, Both HM2 and D4 CAR T cells potently lysed GPC1-positive H8 (**d**), 2B9 (**e**) and T3M4 (**f**) cells without affecting GPC1-negative A431 cells (**c**) after 24 hours of co-culture. **g**, The above culture supernatants from H8 **(d)** and A431 **(c)** at the E:T ratio of 3:1 were harvested to measure IFN-γ, IL-2 and TNF-α secretions via ELISA. n = 3 independent experiments for Fig. 5c-g. $^{**}p < 0.01$, $^{***}p < 0.001$, two-tailed unpaired Student's *t* test. The CAR T cells used in panel c-g were produced using donor

1’s PBMCs. Values represent mean ± SEM. Source data and exact p values are provided in the Source data file.

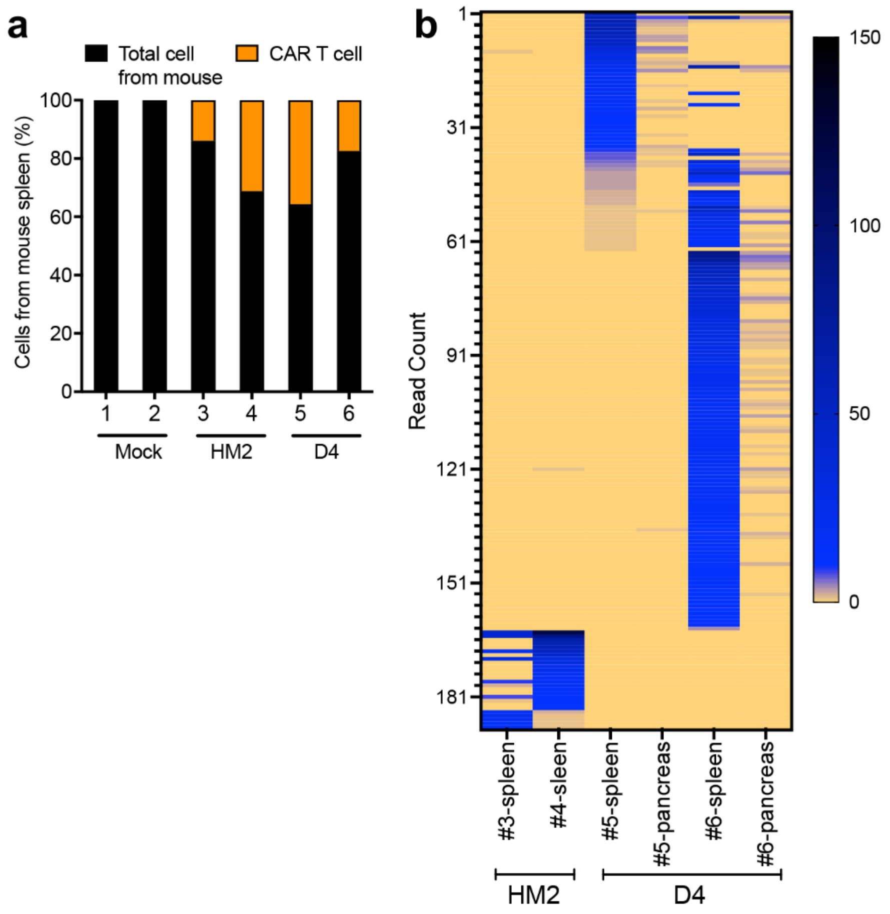

Supplementary Fig. 6: GPC1-targeted CAR T cells exhibit potent antitumor activity in the 2B9 peritoneal dissemination xenograft mouse model. **a**, Detection of CAR vector-positive cells in mouse spleen after 5 weeks of treatment. **b**, Distribution of integration sites in mice treated with HM2 and D4 CAR T cells. The integrated genes were largely shared in T cells recovered from different tissues of the same mouse, while some overlap was also observed in different mice receiving treatment. The CAR T cells used in this study were produced using donor 1's PBMCs. Values represent mean ± SEM. Source data are provided as a Source Data file.

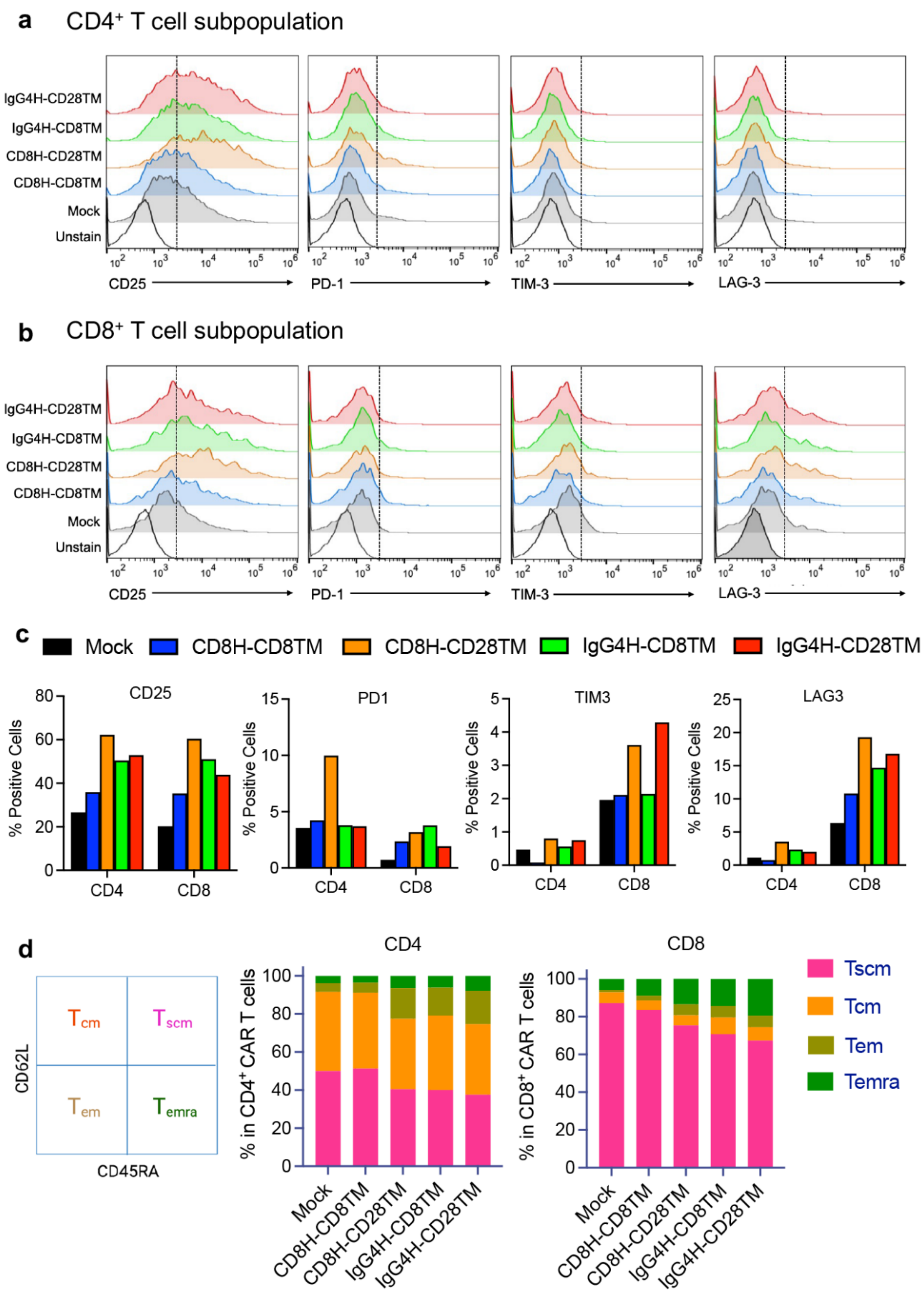

Supplementary Fig. 7: The tonic signaling of D4 CAR T cells with different hinge and transmembrane domain (TM) on day 8 of *ex vivo* expansion. a-b Expression of T-cell

activation marker CD25 and exhaustion markers including PD1, TIM3 and LAG3 after initial activation in $CD4^+$ (**a**) and $CD8^+$ (**b**) D4 CAR T cell subpopulations. **c**, Percentage of exhaustion markers in $CD4^+$ (**a**) and $CD8^+$ (**b**) CAR T cell populations based on **a** and **b**. **d**, Memory T cell phenotypes of D4 CAR T cells with different hinge and TM. Relative proportion of stem cell-like memory (Tscm), central memory (Tcm), effector memory (Tem), and terminally differentiated effector memory (Temra) phenotypes are defined by CD62L, CD45RA and CD95 expression. All experiments in this figure were performed once. The CAR T cells used in this study were produced using donor 2's PBMCs. Source data are provided as a Source Data file.

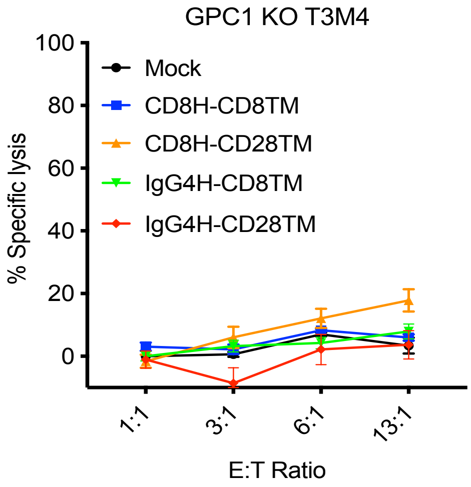

Supplementary Fig. 8: Minimal lysis of GPC1 KO T3M4 cells by any of the D4 CAR T cells. The CAR T cells used in this study were produced using donor 2's PBMCs. n = 3 independent experiments. Values represent mean ± SEM. Source data are provided as a Source Data file.

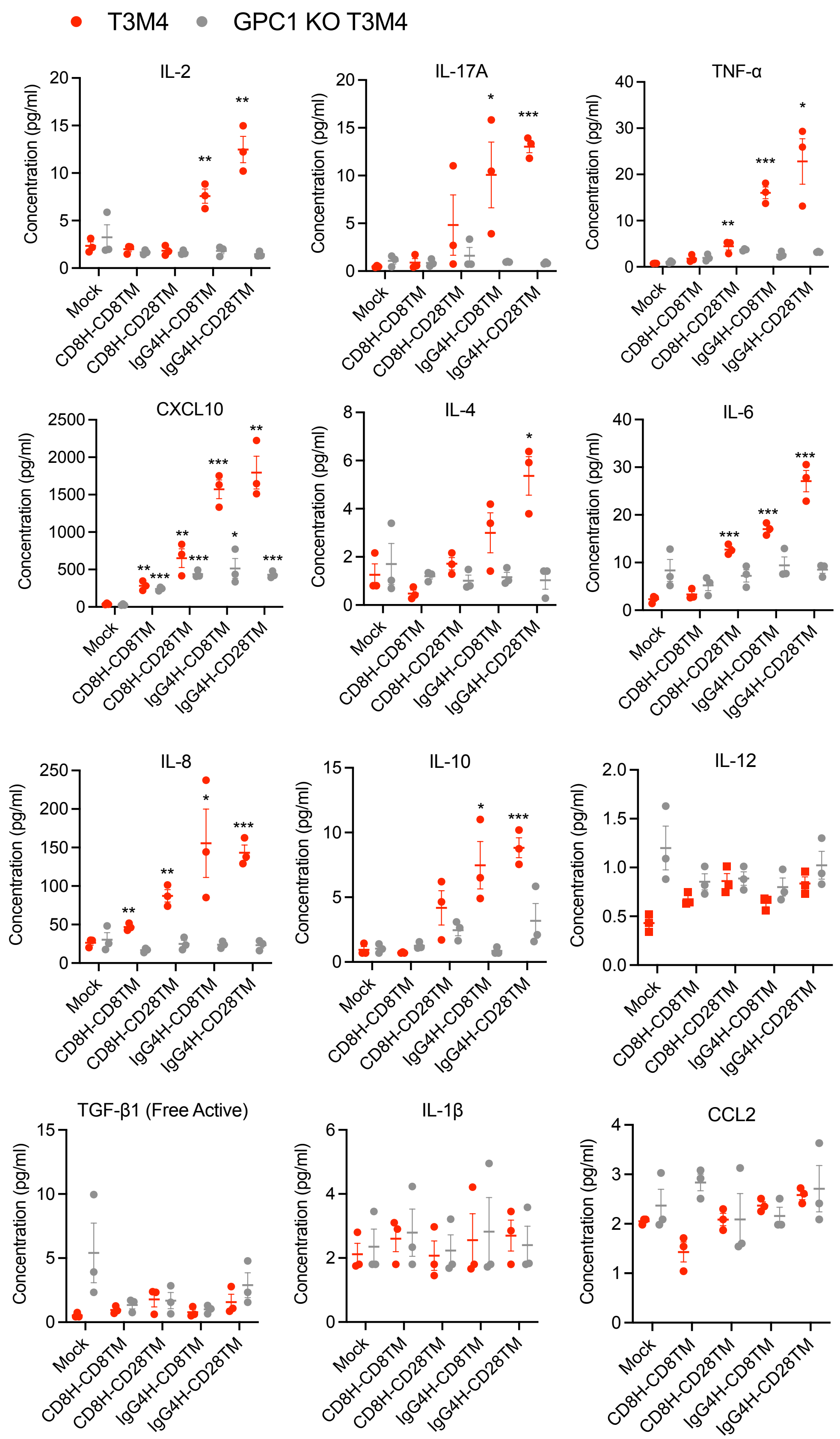
T3M4
GPC1 KO T3M4
IL-2
IL-17A
TNF-α
CXCL10
IL-4
IL-6
IL-8
IL-10
IL-12
TGF-β1 (Free Active)
IL-1β
CCL2
Concentration (pg/ml)
Mock
CD8H-CD8TM
CD8H-CD28TM
IgG4H-CD8TM
IgG4H-CD28TM

Supplementary Fig. 9: Secretion of cytokines and chemokines by D4 CAR T cells with different hinge and TM upon stimulation by GPC1-positive T3M4 and GPC1 KO-T3M4 cells. The CAR T cells used in this study were produced using donor 2's PBMCs. n = 3 independent experiments. *$p < 0.05$, **$p < 0.01$, ***$p < 0.001$, two-tailed unpaired Student's *t* test. Values represent mean ± SEM. Source data and exact p values are provided in the Source data file.

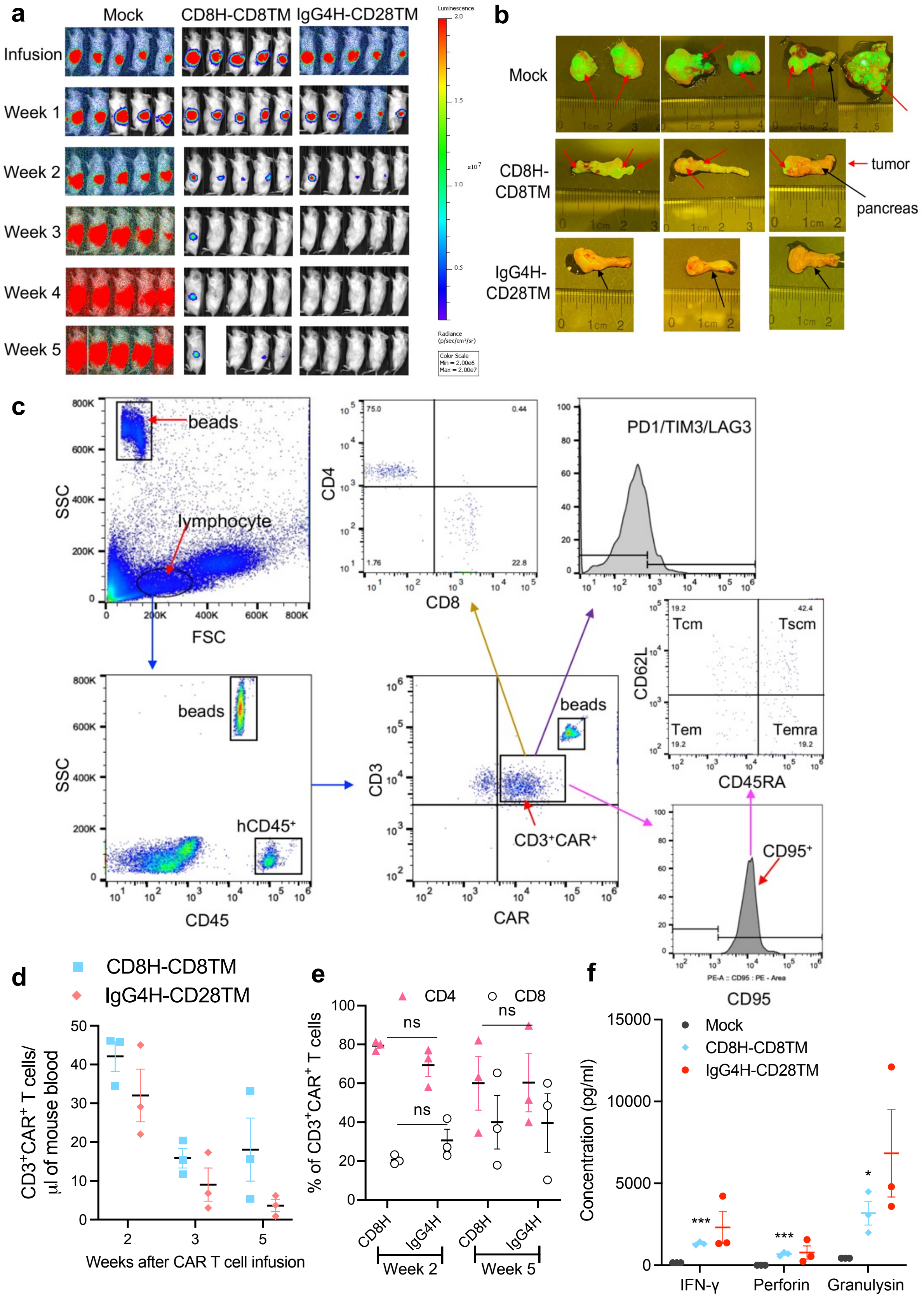
a
Mock
CD8H-CD8TM
IgG4H-CD28TM
Infusion
Week 1
Week 2
Week 3
Week 4
Week 5
b
Mock
CD8H-CD8TM
IgG4H-CD28TM
tumor
pancreas
c
beads
lymphocyte
SSC
FSC
CD4
CD8
PD1/TIM3/LAG3
Tcm
Tscm
Tem
Temra
CD62L
CD45RA
hCD45+
CD45
CD3
CAR
CD3+CAR+
CD95+
CD95
d
CD8H-CD8TM
IgG4H-CD28TM
CD3+CAR+ T cells/ µl of mouse blood
Weeks after CAR T cell infusion
e
CD4
CD8
ns
% of CD3+CAR+ T cells
CD8H
IgG4H
Week 2
Week 5
f
Mock
CD8H-CD8TM
IgG4H-CD28TM
Concentration (pg/ml)
IFN-γ
Perforin
Granulysin

Supplementary Fig. 10: D4-IgG4H-CD28TM CAR T cells demonstrate improved anti-tumor activity in the Panc-1 orthotopic pancreatic xenograft mouse model. a, D4-IgG4H-CD28TM CAR T cells demonstrated enhanced antitumor efficacy compared with D4-CD8H-CD8TM CAR T cells *in vivo*. n = 5 mice/group. **b,** Representative pictures of mouse pancreas and tumors in each group after dissection. Panc-1 cells were tagged with GFP and luciferase. n = 3 mice/group. **c,** Gating strategies of analyzing human T cell CD4/CD8 ratio, exhaustion and phenotype in mouse peripheral blood. **d,** Quantification of absolute $CD3^{+}CAR^{+}$ T cell numbers in mouse peripheral blood during the study period. n = 3 mice/group. **e,** The CD4/CD8 ratios of D4-CD8H-CD8TM and D4-IgG4H-CD28TM CAR T cells in mouse peripheral blood at week 2 and week 5. n = 3 mice/group. ns = $p > 0.05$, two-tailed unpaired Student's *t* test. **f,** Significantly increased secretions of IFN-γ, perforin, and granulysin in the D4-IgG4H-CD28TM CAR group were observed compared with the D4-CD8H-CD8TM CAR group at week 2 post-infusion. n = 3 mice/group. **p* = 0.019, ****p* < 0.001, two-tailed unpaired Student's *t* test. The CAR T cells used in this study were produced using donor 3's PBMCs. Values represent mean ± SEM. Source data and exact p values are provided in the Source data file.

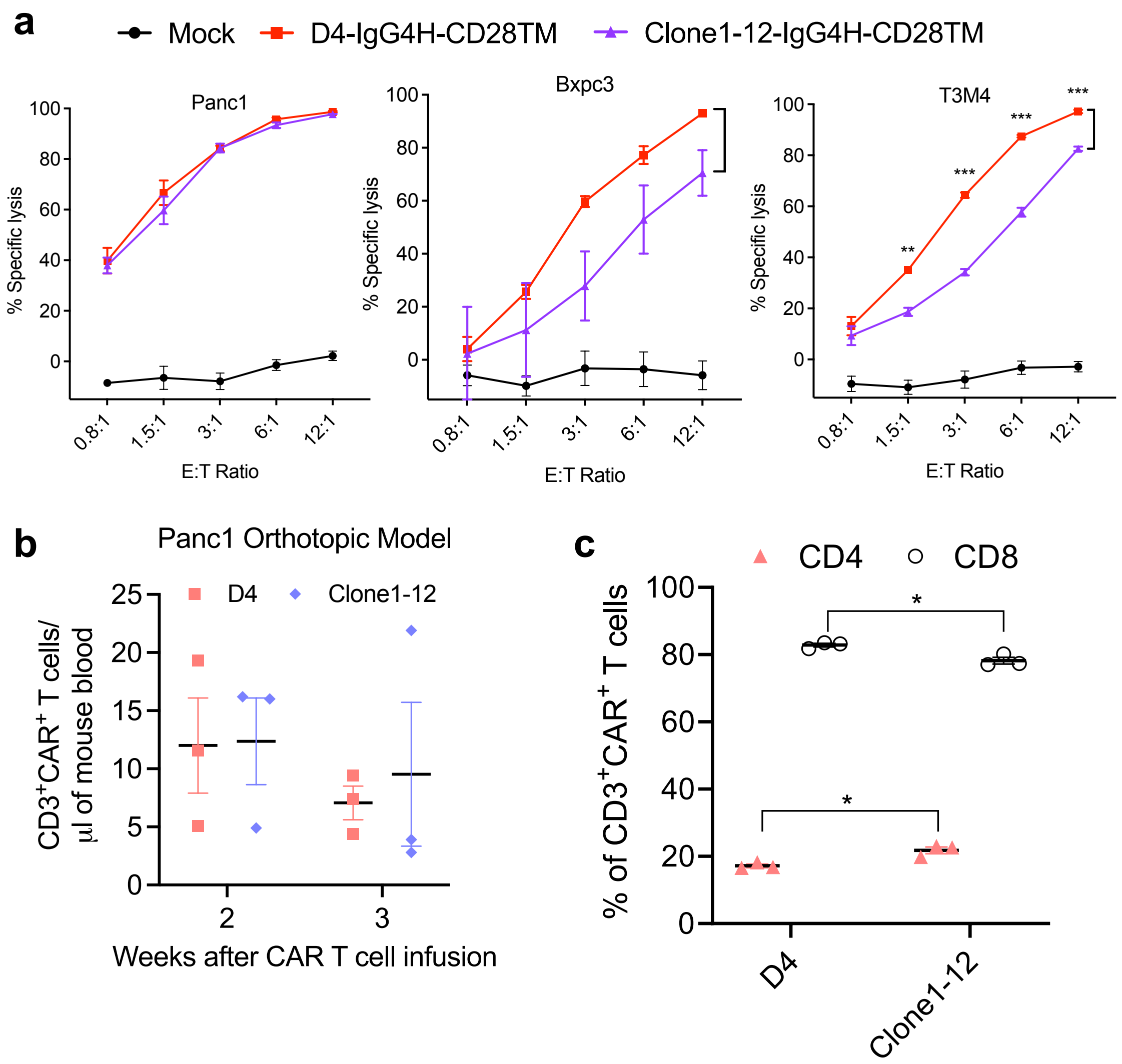

Supplementary Fig. 11: Comparison of D4-IgG4H-CD28TM and clone 1-12-IgG4H-CD28TM CAR T cells *in vitro* and *in vivo*. a, *in vitro* cytolytic activity of both D4-IgG4H-CD28TM and clone 1-12-IgG4H-CD28TM CAR against Panc-1, Bxpc3 and T3M4 cancer cells. n = 3 independent experiments. $^{**}p = 0.0013$, $^{***}p < 0.001$, two-tailed unpaired Student's *t* test. **b,** Quantification of absolute $CD3^+CAR^+$ T-cell numbers in mouse peripheral blood from the Panc1 orthotopic xenograft study in Fig. 5d. n = 3 mice/group. **c,** The CD4/CD8 ratios of D4-IgG4H-CD28TM CAR T and clone 1-12-IgG4H-CD28TM CAR T cells in mouse peripheral blood at week 2. n = 3 mice/group. $^{*}p = 0.015$, two-tailed unpaired Student's *t* test. The CAR T cells used in this study were produced using donor 4's PBMCs. Values represent mean ± SEM. Source data and exact p values are provided in the Source data file.

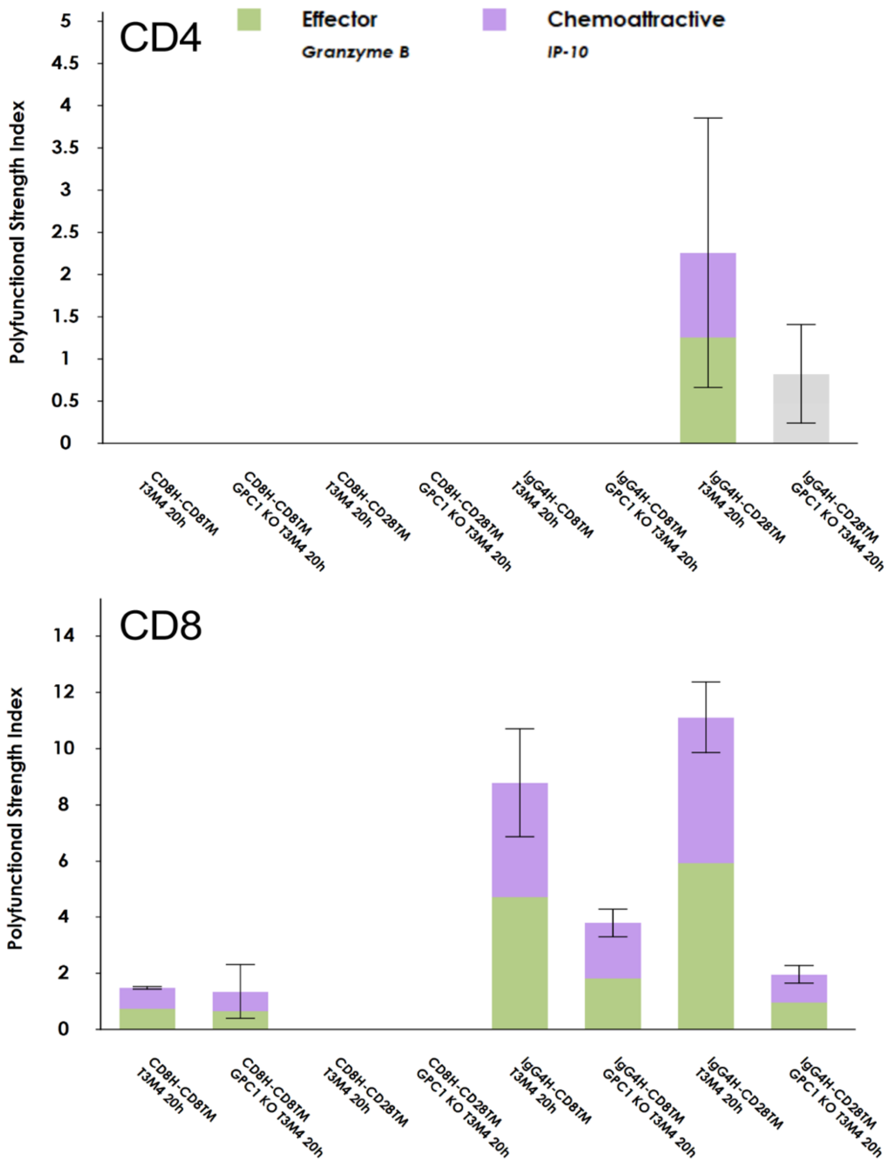

Supplementary Fig. 12: Polyfunctionality of T cells redirected with GPC1. PSI computed for various D4 CAR T cells co-cultured with T3M4 or GPC1 KO T3M4 cells for 20 hours at the single-cell level. The grey column represented the insignificant results below the IsoSpeak software threshold, which is less than 2% of single cells in the group. n = 2 independent experiments. Values represent mean ± SEM. Source data are provided as a Source Data file.

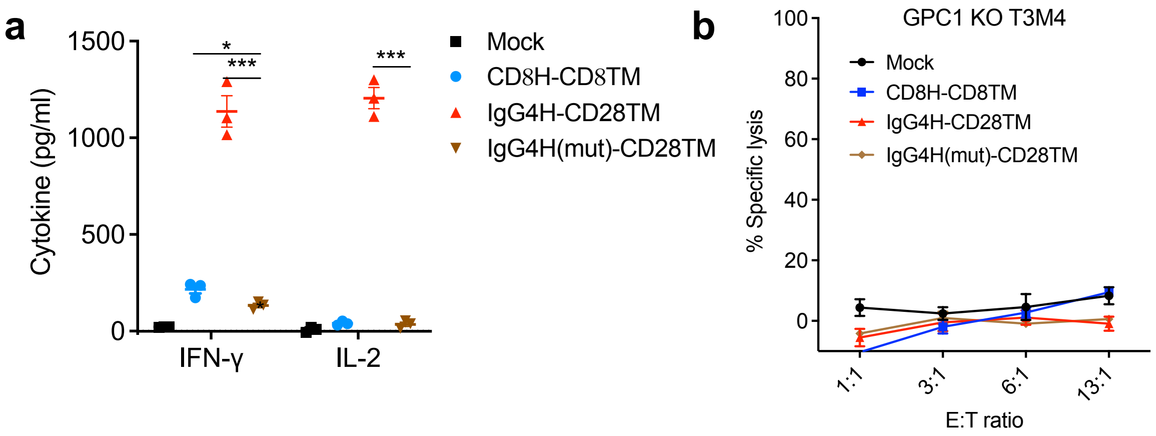

Supplementary Fig. 13: Dimerization is crucial for the potent activity of D4-IgG4H-CD28TM CAR T cells. a, The D4-IgG4 hinge CAR T cells lost the capability to secrete cytokines when both cysteine residues are mutated. **b,** No non-specific killing of various D4 CAR T cells when co-cultured with GPC1 KO T3M4 cells. The CAR T cells used in this study were produced using donor 2's PBMCs. n = 3 independent experiments in Fig. 13. $*p = 0.027$, $***p < 0.001$, two-tailed unpaired Student's t test. Values represent mean ± SEM. Source data and exact p values are provided in the Source data file.

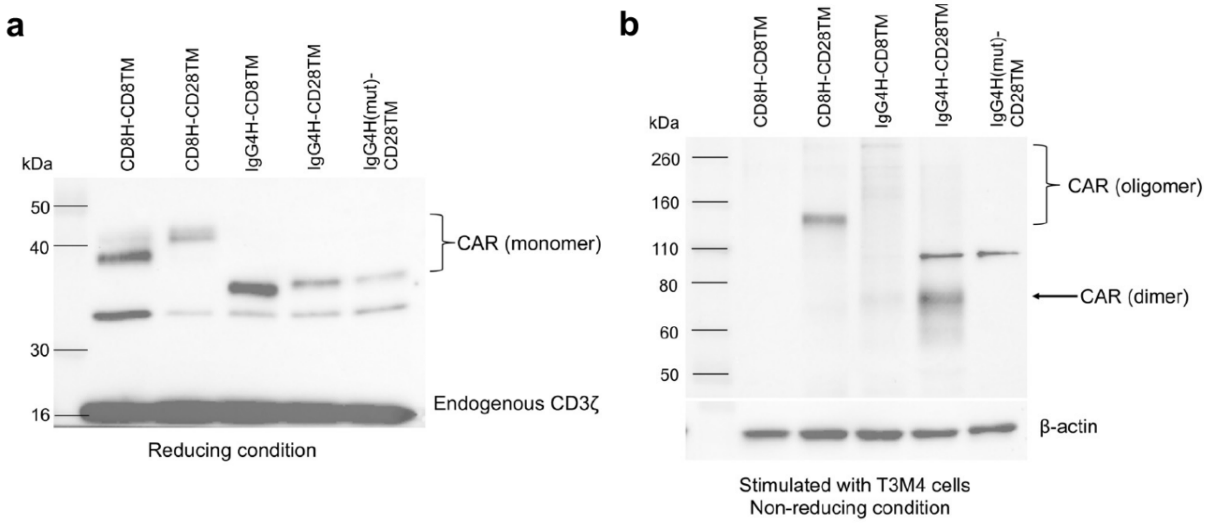

Supplementary Fig. 14: Dimerization of D4-IgG4H-CD28TM CAR after stimulation with T3M4 cells. a, Western blot analysis of CAR expression under reducing condition. n = 1 independent experiment. **b,** Western blot analysis of CAR expression when stimulated with T3M4 cells for 2 hours under non-reducing condition. Membranes were stained with CD3ζ antibody. n = 1 independent experiment. The CAR T cells used in this study were produced using donor 2's PBMCs. Source data are provided as a Source Data file.

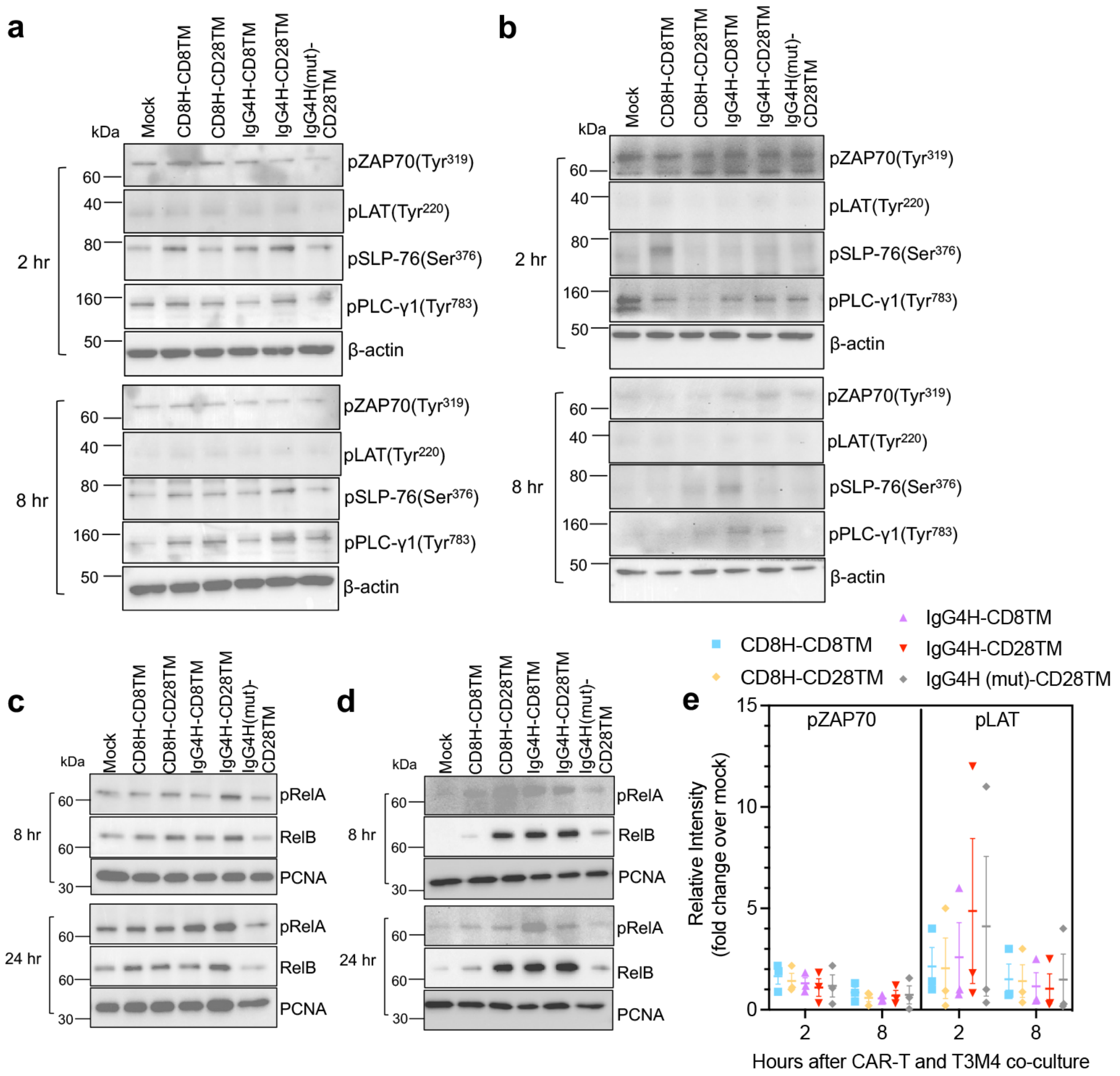

Supplementary Fig. 15: Western blot analysis of T-cell signaling molecules of various D4 CAR T cells upon stimulation with T3M4 cells. a-b, Phosphorylation of T-cell signaling molecules in the cytoplasm. Two separate experiments were performed using T cells prepared from two different donors. **c-d,** Nuclear localization of NF-κB signaling transcription factors in the nucleus. Two separate experiments were performed using T cells prepared from two different donors. **e**, The compiled data of **a-d** and **Fig. 7 f-g** were quantified using Image J and normalized with mock control. n = 3 independent experiments. The CAR T cells used in this study were produced using both donor 2's and donor 4's PBMCs. Values represent mean ± SEM. Source data are provided as a Source Data file.
