# Supplementary material for: The IgG4 hinge with CD28 transmembrane domain improves VHH-based CAR T cells targeting a membrane-distal epitope of GPC1 in pancreatic cancer
Source: Nat Commun. 2023 Apr 8;14:1986. doi: 10.1038/s41467-023-37616-4 (PMC10082787; doi:10.1038/s41467-023-37616-4)
Supplement: Supplementary file 2 — Reporting Summary [file 41467_2023_37616_MOESM2_ESM.pdf]

nature portfolio

Corresponding author(s): Mitchell Ho

Last updated by author(s): March 21, 2023

# Reporting Summary

Nature Portfolio wishes to improve the reproducibility of the work that we publish. This form provides structure for consistency and transparency in reporting. For further information on Nature Portfolio policies, see our Editorial Policies and the Editorial Policy Checklist.

## Statistics

For all statistical analyses, confirm that the following items are present in the figure legend, table legend, main text, or Methods section.

| n/a | Confirmed | |
|---|---|---|
| ☐ | ☒ | The exact sample size (*n*) for each experimental group/condition, given as a discrete number and unit of measurement |
| ☐ | ☒ | A statement on whether measurements were taken from distinct samples or whether the same sample was measured repeatedly |
| ☐ | ☒ | The statistical test(s) used AND whether they are one- or two-sided<br>*Only common tests should be described solely by name; describe more complex techniques in the Methods section.* |
| ☒ | ☐ | A description of all covariates tested |
| ☐ | ☒ | A description of any assumptions or corrections, such as tests of normality and adjustment for multiple comparisons |
| ☐ | ☒ | A full description of the statistical parameters including central tendency (e.g. means) or other basic estimates (e.g. regression coefficient) AND variation (e.g. standard deviation) or associated estimates of uncertainty (e.g. confidence intervals) |
| ☐ | ☒ | For null hypothesis testing, the test statistic (e.g. *F*, *t*, *r*) with confidence intervals, effect sizes, degrees of freedom and *P* value noted<br>*Give P values as exact values whenever suitable.* |
| ☒ | ☐ | For Bayesian analysis, information on the choice of priors and Markov chain Monte Carlo settings |
| ☒ | ☐ | For hierarchical and complex designs, identification of the appropriate level for tests and full reporting of outcomes |
| ☒ | ☐ | Estimates of effect sizes (e.g. Cohen's *d*, Pearson's *r*), indicating how they were calculated |

*Our web collection on statistics for biologists contains articles on many of the points above.*

## Software and code

Policy information about availability of computer code

| | |
|---|---|
| Data collection | SoftMax Pro 7; FortéBio 8.0; LEGENDplex 8.0; Image Lab 2.0; Living Image 4.5.4; BD FACSDiva 6.0; RCutadapt version 3.4; Alevin Salmon version 1.4.0; RossettaFold; AlphaFold; I-Tasser |
| Data analysis | Prism 9; RTsne v. 0.15; DESeq2 v 1.30.1; R pheatmap library v. 1.0.12; Reactome Pathway Browser v 3.7; Yasara v20 |

For manuscripts utilizing custom algorithms or software that are central to the research but not yet described in published literature, software must be made available to editors and reviewers. We strongly encourage code deposition in a community repository (e.g. GitHub). See the Nature Portfolio guidelines for submitting code & software for further information.

## Data

Policy information about availability of data

All manuscripts must include a data availability statement. This statement should provide the following information, where applicable:

- Accession codes, unique identifiers, or web links for publicly available datasets
- A description of any restrictions on data availability
- For clinical datasets or third party data, please ensure that the statement adheres to our policy

Several GPC1 structures including 4ACR (10.2210/pdb4ACR/pdb), 4AD7 (10.2210/pdb4AD7/pdb), 4BWE (10.2210/pdb4BWE/pdb), and 4YWT (10.2210/pdb4YWT/pdb) in the PDB database were used as models in the study. Raw and processed data from the scRNA sequencing experiments are deposited and available in the NCBI's Gene Expression Omnibus (GEO) database under accession code GSE220536 (https://www.ncbi.nlm.nih.gov/geo/query/acc.cgi?acc=GSE220536). The remaining data are available within the Article, Supplementary Information or Source Data files.

## Human research participants

Policy information about studies involving human research participants and Sex and Gender in Research.

| | |
|---|---|
| Reporting on sex and gender | The information about the sex and gender of healthy donors was not collected. |
| Population characteristics | Sex, age or any identifiable information of healthy donors was not collected. |
| Recruitment | Human peripheral blood samples from healthy donors were provided by the NIH Blood Bank (approved by the NIH Clinical Center Department of Transfusion Medicine). Informed written consent was obtained from all healthy donors for the use of their blood samples for laboratory research purposes by NIH researchers. Pancreatic tumor tissue microarray was purchased from US Biomax (Rockville, USA). |
| Ethics oversight | The use of deidentified human specimens was determined to be the NIH Institutional Review Board (IRB) exempt. Because the specimens or data were not collected specifically for our study and no one on our study team has access to the subject identifiers linked to the specimens or data, our study is not considered as human subjects research. |

Note that full information on the approval of the study protocol must also be provided in the manuscript.

# Field-specific reporting

Please select the one below that is the best fit for your research. If you are not sure, read the appropriate sections before making your selection.

☒ Life sciences ☐ Behavioural & social sciences ☐ Ecological, evolutionary & environmental sciences

For a reference copy of the document with all sections, see nature.com/documents/nr-reporting-summary-flat.pdf

# Life sciences study design

All studies must disclose on these points even when the disclosure is negative.

| | |
|---|---|
| Sample size | Sample sizes for in vivo experiments, e.g. treatment cohorts, were used on the basis of ensuring results obtained were of a representable quantity. 5 or 6 mice per group were used to ensure statistical power. For in vitro studies, sample size of at least 3 was used in each experiment. Sample size and number of independent experiments are stated in the figure legends. |
| Data exclusions | No data points was excluded from analysis. |
| Replication | Three replicates were successfully performed to ensure data reproducibility. |
| Randomization | Once mice were confirmed to carry tumors, they were randomized into different treatment arms/cages. Cell culture experiments were also randomly assigned to different groups. |
| Blinding | Investigators were blind for randomization. |

# Reporting for specific materials, systems and methods

We require information from authors about some types of materials, experimental systems and methods used in many studies. Here, indicate whether each material, system or method listed is relevant to your study. If you are not sure if a list item applies to your research, read the appropriate section before selecting a response.

## Materials & experimental systems

| n/a | Involved in the study | |
|---|---|---|
| | x | Antibodies |
| | x | Eukaryotic cell lines |
| x | | Palaeontology and archaeology |
| | x | Animals and other organisms |
| x | | Clinical data |
| x | | Dual use research of concern |

## Methods

| n/a | Involved in the study | |
|---|---|---|
| x | | ChIP-seq |
| | x | Flow cytometry |
| x | | MRI-based neuroimaging |

# Antibodies

Antibodies used

Goat anti-mouse IgG conjugated with phycoerythrin (PE) (Jackson ImmunoResearch, Cat#115-116-072) at 1:200 dilution.
Goat anti-mouse IgG conjugated with allophycocyanin (APC) (Jackson ImmunoResearch, Cat#115-136-072) at 1:200 dilution
anti-FLAG antibody conjugated with APC (Biolegend, Cat#637308) at 1:100 dilution
anti-EGFR human monoclonal antibody cetuximab (Erbitux) at 1 μg/ml
goat anti-human IgG conjugated with PE (Jackson ImmunoResearch, Cat#109-116-097) at 1:200 dilution
BV421 CD45 (Biolegend, Cat#368522, 5 μl/sample)
BV605 CD3 (Biolegend, Cat#317322, 5 μl/sample)
APC-H7 CD4 (BD Bioscience, Cat#560158, 5 μl/sample)
PerCP-Cy5.5 CD8 (Biolegend, Cat#344710, 5 μl/sample)
goat-anti-human IgG conjugated with Alexa Fluor 488 (Jackson ImmunoResearch, Cat#109-546-097) at 1:200 dilution
PE PD1 (Biolegend, Cat# 379210, 5 μl/sample)
PE-Cy7 TIM3 (Biolegend, Cat#345014, 5 μl/sample)
APC LAG3 (Biolegend, Cat# 369212, 5 μl/sample)
BV421 CD45RA (BD Bioscience, Cat# 562885, 5 μl/sample)
APC CD62L (BD Bioscience, Cat# 566791, 5 μl/sample)
PE CD95 (Biolegend, Cat# 305608, 5 μl/sample)
PE CD25 (Thermo Fisher Scientific, Cat#12-0257-42, 5 μl/sample)
PE CD27 (Thermo Fisher Scientific, Cat#12-0279-42, 5 μl/sample)
PE CD127 (Thermo Fisher Scientific, Cat#12-1278-42, 5 μl/sample)
HM2 was used to detect GPC1 expression at 1 μg/ml
anti-CD3ζ antibody (Santa Cruz Biotechnology, Cat#sc-166435, 1:200 dilution)
anti-ZAP70-pTyr319 (Cell Signaling Technology, Cat#2701S, 1:500 dilution)
anti-LAT-pTyr220 (Cell Signaling Technology, Cat#3584S, 1:500 dilution)
anti-SLP-76-pSer376 (Cell Signaling Technology, Cat#14745S, 1:500 dilution)
anti-PLC-gamma1-pTyr783 (Cell Signaling Technology, Cat#2821S, 1:500 dilution)
anti-RelA/p65-pSer536 (Cell Signaling Technology, Cat#3033S, 1:500 dilution)
anti-RelB (Cell Signaling Technology, Cat#10544S, 1:500 dilution)
anti-beta-catenin (Cell Signaling Technology, Cat#9582S, 1:1000 dilution)
anti-beta-actin (Cell Signaling Technology, Cat#8457S, 1:2000 dilution)
GAPDH (Cell Signaling Technology, Cat#5174S, 1:5000 dilution)
anti-PCNA (Cell Signaling Technology, Cat#2586S, 1:1000 dilution)
Anti-Wnt3a (Abcam, Cat#ab28472, 1:100 dilution)

Validation

Commercial antibodies are all validated by the vendors and the validation data is available on the vendors' website.

# Eukaryotic cell lines

Policy information about cell lines and Sex and Gender in Research

Cell line source(s)

The A431 (originally from ATCC #CRL-1555) and HEK-293T (originally from ATCC #CRL-3216) cell lines were obtained from Dr. Ira Pastan at the NCI. H8, a transfected A431 cell line stably expressing human GPC1, was made in our lab. HEK293 SuperTopflash stable cell line was obtained from Dr. Jeremy Nathans at the Johns Hopkins University. Pancreatic cancer cell lines including T3M4, Aspc1 (originally from ATCC #CRL-1682), Bxpc3 (originally from ATCC #CRL-1687), Colo357, and SU8686 (originally from ATCC #CRL-1837) were obtained from Dr. Udo Rudloff at the NCI. Miapaca-2 (originally from ATCC #CRL-1420) and Panc-1 (originally from ATCC #CRL-1469) pancreatic cancer cell lines were obtained from Dr. Perwez Hussain at the NCI. KLM1 pancreatic cancer cell line was obtained from Dr. Christine Alewine at the NCI. The hTERT-HPNE cell line (originally from ATCC #CRL-4023) was also obtained from Dr. Perwez Hussain at the NCI. A431, H8, KLM1, 2B9, T3M4, Bxpc3, and Panc-1 cell lines were engineered to express luciferase (Luc) and GFP in our lab.

Authentication

STR profiling was used for cell line authentication.

Mycoplasma contamination

All cell lines tested negative for mycoplasma contamination.

Commonly misidentified lines
(See ICLAC register)

No commonly misidentified lines were used in the study.

## Animals and other research organisms

Policy information about studies involving animals; ARRIVE guidelines recommended for reporting animal research, and Sex and Gender in Research

| | |
|---|---|
| Laboratory animals | 5-7 weeks of female NOD/SCID/IL-2Rgcnull (NSG) mice were used in this study. Mice were housed under a standard 12:12 h light/dark cycle with a standard food and water. |
| Wild animals | No wild animals were used in this study. |
| Reporting on sex | The findings are based on tumor models in female mice. We have indicated the sex in the Abstract section. |
| Field-collected samples | No field-collected samples were used in this study. |
| Ethics oversight | The mouse experiments were conducted under the protocol (LMB-059) approved by the Institutional Animal Care and Use Committee at the NIH. |

Note that full information on the approval of the study protocol must also be provided in the manuscript.

## Flow Cytometry

### Plots

Confirm that:

- [x] The axis labels state the marker and fluorochrome used (e.g. CD4-FITC).
- [x] The axis scales are clearly visible. Include numbers along axes only for bottom left plot of group (a 'group' is an analysis of identical markers).
- [x] All plots are contour plots with outliers or pseudocolor plots.
- [x] A numerical value for number of cells or percentage (with statistics) is provided.

### Methodology

| | |
|---|---|
| Sample preparation | Samples were stained in 5% BSA in PBS with different antibody dilutions. |
| Instrument | SONY ID7000, LSR-Fortessa |
| Software | FlowJo |
| Cell population abundance | Cell population abundance was determined using counting beads. |
| Gating strategy | Live cells were gated from FSC/SSC plot, then CD45+ lymphocytes were gated on live cells. Next, CD3+ cells were gated on CD45+ lymphocytes. T cell exhaustion and differentiation were analyzed in the CD3+CD45+ T cell population. |

- [x] Tick this box to confirm that a figure exemplifying the gating strategy is provided in the Supplementary Information.
